# Enablers and barriers for policymaker engagement in health research from the perspective of policymakers: a scoping review

**DOI:** 10.1136/bmjopen-2025-099720

**Published:** 2025-08-21

**Authors:** Lorena Guerrero-Torres, Anas Ismail, William Savedoff, Kabir Sheikh, Meike Schleiff

**Affiliations:** 1 Alliance for Health Policy and Systems Research, World Health Organization, Geneva, Switzerland; 2 Social Insight, Washington, Maine, USA; 3 Global Business School for Health, University College London, London, UK

**Keywords:** Health policy, Decision Making, Systematic Review

## Abstract

**Abstract:**

**Objectives:**

Over the past two decades, initiatives promoting research-policy engagement have increased broadly and in health. Numerous factors influencing the engagement of policymakers in research have been described primarily from the perspective of researchers. This scoping review aimed to identify the enablers and barriers to policymaker engagement across the research process from the perspective of policymakers.

**Design:**

Scoping review following the Joanna Briggs Institute Methods Manual for scoping reviews.

**Data sources:**

MEDLINE, Cochrane Library, Social Policy and Practice, Campbell Collaboration, Health Systems Evidence and World Bank e-Library, supplemented by grey literature from Google Scholar, WHO Global Index Medicus and VHL Regional Portal.

**Eligibility criteria:**

We included English language studies published after 2007 that involved policymakers at national or subnational levels who were actively engaged in research at any stage. We excluded studies which did not include policymakers, where engagement was passive, or perspectives were marginal or not clearly outlined.

**Data extraction and synthesis:**

After screening and full-text review, we extracted and coded data using MAXQDA Plus 24. We conducted thematic analysis, categorising findings as enablers or barriers into three levels: individual, organisational and contextual/system. Findings were iteratively reviewed and refined by the research team.

**Results:**

We screened 5384 titles and abstracts, reviewed 59 full-text documents and included 30 articles for analysis. Most studies were published after 2016 and were focused on policymaker engagement at the national level. Organisational factors were the most frequently reported influences on engagement of policymakers in research across different contexts. The most frequent enablers mentioned in the literature were (1) the institutionalisation of partnerships, initiatives and having formal agreements; (2) defining goals, roles, responsibilities and conflict resolution mechanisms; (3) researchers providing practical and expert advice to policymakers; (4) leveraging networks; and (5) having supportive institutions. The most frequent barriers were (1) the lack of regulations, infrastructure, funding and communication channels to support engagement; (2) the lack of skills of researchers to understand policymaking processes and work in collaboration with policymakers; and (3) the mismatch in priorities, values, perspectives and expectations.

**Conclusions:**

Our study highlights the role of institutional support, widespread collaboration opportunities and the interconnected nature of these factors within the research-policy ecosystem.

**Study registration:**

Open Science Framework (https://osf.io/ynr78/).

**Strengths and limitations of this study**

- This scoping review followed Joanna Brigs Methods Manual for Scoping Reviews, ensuring methodological replicability and transparency.
- Conducted a thorough literature search across several peer-reviewed literature and complemented with grey literature, which helped capture a wide range of studies
- Applied a structured approach and rigorous methodology to limit bias throughout the study and eliciting the perspectives of policymakers as presented in the included literature.
- The study only included articles published in English which might have introduced bias by missing relevant research published in other languages.
- We did not appraise the methodological quality of included studies; however, this is consistent with the scoping review design and results of this study should be interpreted as an overview of the evidence.

## Introduction

 With multiple crises ongoing and shrinking budgets for health and research, improving the relevance of research for evidence-based decision-making is needed. Research-policy collaboration and partnerships have been recognised as effective for advancing evidence-informed policy.^1^ However, policymaking is a complex and non-linear process influenced by the context, ideologies and values, where evidence is one of many inputs. Moreover, the policy process is country-specific and depends on policy structures and mechanisms.^1^,^3^ Although researchers lack the power to redesign the policymaking process, they can improve the way they engage with policymakers, adapt to their environments to identify relevant policymakers, identify opportunities to influence policy and frame problems in a convincing way.^4^

Vast research exists about the value of engaging policymakers in research processes. For instance, it is known that including knowledge end-users in the research process reduces research waste.^5^ Numerous initiatives and approaches for engaging policymakers in health research have been implemented in different contexts and settings, including co-production and integrated knowledge translation (IKT).^6^,^9^ Although these strategies are known to improve the quality and relevance of research, increase its utilisation, increase accountability and transparency to research funders, and empower and give ownership to policymakers,^2 10^ several factors can influence the engagement of policymakers in the research process.

Studies focusing on the factors in successful engagement include early engagement in the process, detailing expectations and roles clearly from the beginning, maintaining ongoing relationships to build trust and credibility and being sensitive to time constraints.^6^ Yet, most of this literature centres around the perspective of researchers and the research community. This researcher-centred approach often overlooks the diverse challenges that policymakers face in their complex and dynamic environment, which can shape how they engage with and use research. In conducting this study, we attempt to fill this gap in the literature to inform the development of more effective strategies that facilitate meaningful collaboration and ensure that research is responsive to policy needs.

The objective of this study is to identify the enablers and barriers to policymaker engagement across the research process from the perspective and experience of policymakers. The scoping review achieves this by assessing frameworks and evidence in studies that contain policymakers’ perspectives. A preliminary search for existing systematic and scoping reviews on the topic has been conducted in the Cochrane Database of Systematic Review and JBI Evidence Synthesis, and no reviews have been found.

## Methods

To fulfil our objective, we conducted a scoping review following the Joanna Briggs Institute Methods Manual for scoping reviews.^11^ This methodology was selected to explore and map the diverse knowledge base on the topic^11^ and identify particular factors.^12^ The findings are reported following the Preferred Reporting Items for Systematic Reviews and Meta-Analyses extension for Scoping Reviews (PRISMA-ScR) Checklist (online supplemental appendix A).^13^ The full protocol is registered in the Open Science Framework (https://osf.io/ynr78/) and can be found in the online supplemental appendix B.

### Eligibility criteria

Our eligibility criteria followed the Population, Intervention, Comparison and Outcome (PICO) framework and are detailed in the online supplemental appendix B. Our review focused on studies that captured the enablers and barriers to policymaker engagement in research from the perspective of policymakers, including qualitative studies. This emphasis on policymaker perspectives guided the exclusion of studies where the perspectives or views of policymakers were marginal, not clearly outlined or presented together with the views of other stakeholders. In studies where researchers’ and policymakers’ perspectives were presented separately, we extracted data specifically from policymakers, not researchers. Studies reporting general insights on evidence use but lacking perspectives relevant to the engagement of policymakers in research were excluded.

Policymakers are a heterogeneous group whose roles, responsibilities and influence vary depending on governance structures, health system organisation and political context. For the purposes of this review, we included studies that involved policymakers, decision-makers or regulators at any level (national or subnational). We adopted the definition of Deverka *et al* for policymakers and regulators: ‘individuals and organisations that create, monitor and oversee policies or regulations of healthcare-related issues, such as federal, state and local government agencies, medical and professional organisations and clinical guidelines developers’.^14^ This definition acknowledges that policy and decision-making not only occur within government but also in other professional bodies and recognises the diversity of these actors across national and subnational contexts. From here on, we use ‘policymakers’ to refer to policymakers, decision-makers and regulators.

For a study to be included, policymakers had to be actively engaged in research at any point (topic selection, conceptualisation, data collection, etc.) through any engagement method (meetings, workshops, etc.). We included all study designs reporting factors influencing policymaker engagement in the research process. We excluded studies that did not include policymakers or where engagement was passive (such as merely presenting research findings). We excluded editorials and commentaries and did not restrict settings.

### Data sources and search strategy

The literature search included MEDLINE, Cochrane Library, Social Policy and Practice, Campbell Collaboration, Health Systems Evidence and the World Bank e-Library. We supplemented this search with grey literature sources (Google Scholar, WHO Global Index Medicus and VHL Regional Portal). Multiple search strategies were developed, reflecting the different approaches to identify the type of evidence needed to reach the study objectives.

The peer-review search was executed between 25 April and 5 May 2023, while the grey literature search was executed on 7 June 2023. The online supplemental appendix C details the search strategy and results. In Google Scholar, we included only the first 998 results of the search for title and abstract screening. We restricted our search to articles in English, published from 2007 onwards, coinciding with the publication of the ‘Sound Choices’ flagship report of the Alliance for Health Policy and Systems Research,^3^ which catalysed efforts to integrate research into decision-making processes and emphasised the importance of engaging policymakers.

### Data extraction and analysis

We downloaded RIS files from the data sources and imported all records into Rayyan, where we removed duplicated records and recorded screening decisions. Two independent reviewers (LGT and AI) screened the titles and abstracts of the remaining articles (n=5384). Fifty-nine articles were included for full-text review. The lead author (LGT) reviewed the full text of all 59 articles. AI, WS and MS were the second full-text reviewers. A third reviewer resolved disagreements. We used Microsoft Excel to summarise the decisions.

We produced a standardised extraction form using Microsoft Excel, including the title, publication year, author, geographic region or country of focus, article type, methods, characteristics of the initiative/intervention, population, participants included and findings for the thematic analysis. We extracted data from the methods and result sections of articles and from the discussions where authors noted their interpretation of results. The initial codes were developed deductively by combining three main frameworks: the SPIRIT Action Framework,^15^ the Evidence needs of health system decision-makers^16^ and the conceptual framework of evidence-informed health policymaking.^3^ The combination of frameworks aims to explain empirical data using insights from multiple frameworks. This approach removes assumptions that one framework is better and enables a greater understanding of the topic.^17 18^

Two reviewers (LGT and WS) conducted a pilot test and extracted data using the initial extraction table. Reviewers then met to discuss the extraction table and codes and refine them. We used MAXQDA Plus 24 software to code, organise and analyse the data. To systematically code the data, we developed a codebook with detailed definitions. LGT, MS and WS discussed the codebook, proposed refinements to avoid redundancy and agreed on modifications. A final codebook version can be found in the online supplemental appendix D.

Once all extractions and coding were completed, the lead author categorised all engagement factors as enablers or barriers. Barriers were defined as factors that hinder the engagement of policymakers in the research process. Conversely, enablers are factors that may help policymakers to engage in the research process. The coding strategy is illustrated in the online supplemental appendix E. The research team discussed the initial findings from this analysis, which was an iterative and reflective process.^19^ We did not critically appraise the included sources of evidence.

We selected the conceptual framework for understanding and assessing research-policy partnerships^20^ as the most appropriate to organise and report the enablers and barriers. This framework identifies three components of research-policy partnership: influencing factors, partnership processes and effects of partnership. The processes are the stages of the partnership: from establishing the partnership to supporting or adjusting it to its dissolution. The key influences are the individual, organisational and contextual factors that enable or constrain the partnership. The framework acknowledges that these factors are interrelated. The effects are the changes and implications arising from the partnership and may be intended and unintended. Although our study reports on different types and models of researcher-policymaker engagements, not only partnerships, the framework was used to organise the enablers and barriers across the three levels of key influencers (individuals, organisational and contextual/system).

### Patient and public involvement

It was not appropriate to involve patients or the public in the design, conduct, reporting or dissemination plans of our research.

## Results

### Literature search

**Figure 1:**
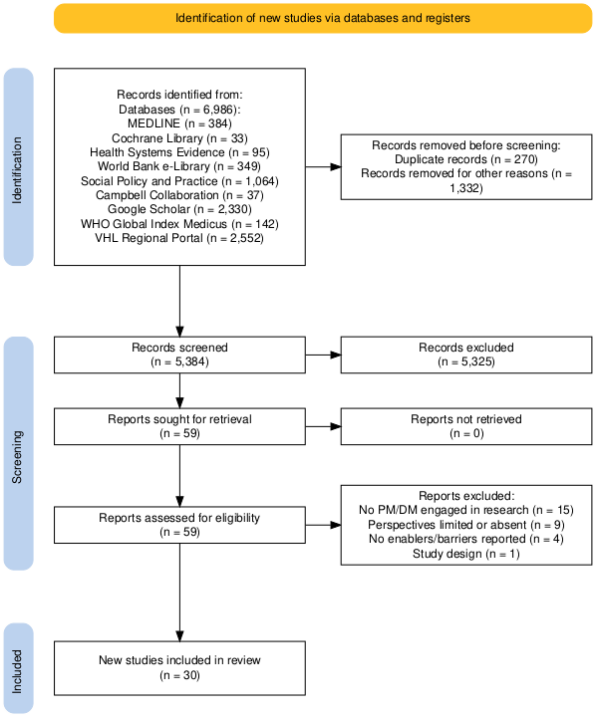
Preferred Reporting Items for Systematic Reviews and Meta-Analyses (PRISMA) flow chart of included studies.

After screening 5384 titles and abstracts and reviewing 59 full-text documents, 30 articles fulfilled our eligibility criteria (figure 1). The full citations are included in the online supplemental appendix F. Fifteen documents were excluded because they did not discuss policymaker engagement in the research process, nine were excluded because the perspectives of policymakers were absent or limited, and four did not include factors influencing the engagement of policymakers in research. No additional relevant documents were identified by scanning the reference lists of the included articles.

### Characteristics of the included articles (n=30)

**Figure 2:**
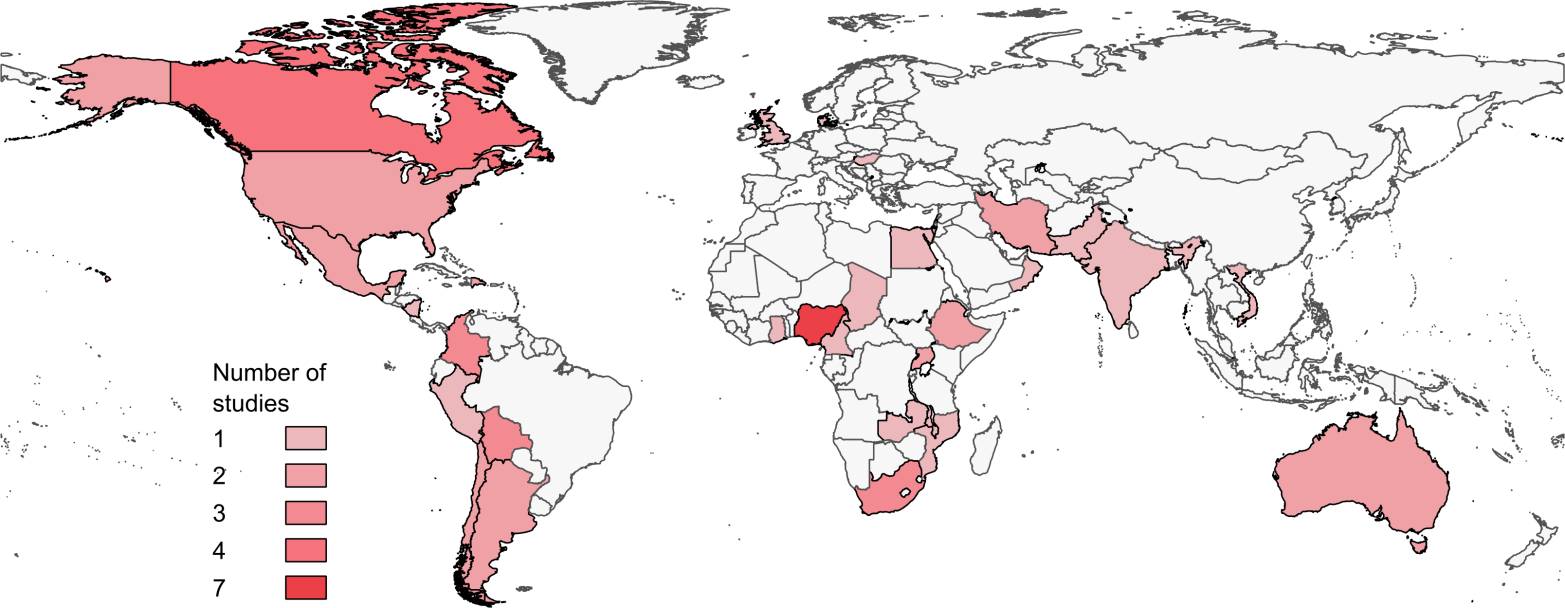
Heat map of included articles* by geographic focus. *The only study not included is Shroff ZC *et al*,^49^ which involved perspectives of policymakers from 24 Ministries of Health in low- and middle-income countries.

Characteristics of the literature are displayed in table 1. Most references were published from 2017 onwards (n=23, 77%). The remainder of the returns were published from 2009 to 2016. The first authors were most commonly from North America (n=10), Europe (n=9) and Africa (n=6). Most articles focused on a single country (n=21, 70%), with Nigeria being the most common setting (n=7, 23%), followed by Canada (n=4, 13%) (figure 2). Nine articles focused on more than one country, six presenting data from different countries and regions, three focusing on countries in the Americas and one on the Afro region.

**Table 1:** Included studies

No	Author, year	Study settings	Point(s) of engagement	Method of engagement
1	Abubakar *et al*^47^ 2021	National (Nigeria)	Priority setting, conceptualisation, KPP, uptake and evaluation	Co-production (regular meetings, calls and communications)
2	Badakhshan *et al*^33^ 2018	National (Iran)	Priority-setting	Not described
3	Bowen *et al*^39^ 2019	National (Canada)	Not described	Not described
4	Cambe *et al*^32^ 2022	National (Mozambique)	Priority setting, conceptualisation, uptake and evaluation	Not described
5	Ellen *et al*^48^ 2018	National (Israel)	Not described	Not described
6	Gollust *et al*^36^ 2017	National (United States)	Not described	Not described
7	Haynes *et al*^31^ 2011	National (Australia)	Not described	Meetings, committees, advisory groups and stakeholder forums
8	Hyder *et al*^37^ 2011	National (Argentina, Egypt, Iran, Malawi, Oman, Singapore)	Not described	Not described
9	Jessani *et al*^30^ 2020	National (USA)	Not described	Not described
10	Khan *et al*^21^ 2014	Subnational (Ontario, Canada)	Priority setting, conceptualisation, uptake and evaluation	Researcher-policymaker partnership with monthly meetings and informal communications (eg, email, phone)
11	Langlois *et al*^45^ 2019	National (Argentina, Chile, Peru, Saint Lucia); subnational (Argentina, Bolivia, Brazil, Chile, Colombia, Mexico)	Conceptualisation, data collection, KPP and uptake	Decision-maker-led projects (meetings, training sessions, communications)
12	Langlois *et al*^40^ 2016	National (South Africa, Cameroon); subnational (Mexico, Nicaragua)	Conceptualisation, KPP, and uptake	Policy BUDDIES: one-on-one meetings and dialogues. CoP: workshops and online communities of peers
13	Loncarevic *et al*^2^ 2021	National (Denmark)	Conceptualisation, data analysis and uptake and evaluation	Not described
14	Mancuso *et al*^38^ 2021	Subnational (Chad, Ethiopia, Nigeria, Uganda, Vietnam, India, Pakistan)	Conceptualisation, data collection, KPP and uptake	Decision-maker-led projects (meetings, conferences, policy dialogues, and working groups)
15	Mansilla *et al*^57^ 2017	National (Chile)	Uptake of research mostly	Rapid response service (preparing rapid evidence syntheses), workshops
16	Mendell and Richardson^22^ 2021	Subnational (British Columbia, Canada)	Priority setting, conceptualisation, KPP, uptake and evaluation	Meetings and events, phone calls, email updates and presentations
17	Mihalicza *et al*^23^ 2018	National (Hungary)	Uptake of research	In-person meetings (conferences, roundtables, forums, advisory boards)
18	Mijumbi-Deve *et al*^42^ 2022	National (Ethiopia, Lebanon and South Africa)	Priority setting, conceptualisation, data synthesis, KPP and uptake	Meetings and emails
19	Mirzoev *et al*^20^ 2012	National (Ghana, South Africa, Uganda and Zambia)	Priority setting, conceptualisation, data collection and data analysis	Diverse partnerships (Uganda (formal and informal communications); South Africa (formal communication))
20	Onwujekwe *et al*^46^ 2019	Subnational (Enugu and Anambra states, Nigeria)	Priority setting and uptake of research	Document sharing, conferences, workshops and seminars
21	Onwujekwe *et al*^44^ 2020	Subnational (Enugu and Anambra states, Nigeria)	Not described	Workshops
22	Shroff *et al*^49^ 2017	Global	Not described	Not described
23	Smith *et al*^58^ 2009	Subnational (British Columbia, Canada)	Priority setting	Forums
24	Uneke *et al*^35^ 2017	National (Nigeria)	Uptake of research	In-person meeting
25	Uneke *et al*^41^ 2017	National (Nigeria)	Not described	Two-way secondment programme between the University and the MOH
26	Uzochukwu *et al*^34^ 2016	Subnational (Enugu, Anambra and Lagos states, Nigeria)	Mostly priority-setting and conceptualisation	Policy maker-initiated and researcher-initiated research (meetings, workshops)
27	Van der Graaf *et al*^43^ 2017	National (England)	Priority-setting, conceptualisation and uptake	Not described
28	Varallyay *et al*^25^ 2020	National (Dominican Republic); subnational (Bolivia and Colombia)	Conceptualisation, data collection and synthesis, KPP and uptake	Decision-maker-led research projects
29	Varallyay *et al*^24^ 2022	National (Dominican Republic); subnational (Bolivia and Colombia)	Conceptualisation, data collection and synthesis, KPP and uptake	Decision-maker-led research projects
30	Williamson *et al*^29^ 2019	Subnational (New South Wales, Australia)	Conceptualisation, KPP, uptake and evaluation	Partnerships with different models (co-production, policymaker-led research)

CoP, community of practice; KPP, knowledge product preparation; MOH, Ministry of Health.

As described in table 1, the study settings of most articles were national (n=18, 60%) and subnational (n=8, 27%). Most studies (n=29, 97%) included in this review employed descriptive designs relying on qualitative or mixed-methods, such as interviews, surveys and document reviews. The number of policymakers included as participants in the studies varied widely from 7 to 600. Half of the studies engaged policymakers in several phases of the research process and used different engagement methods.

### Frameworks on the engagement of policymakers in research

Seven included articles mentioned six frameworks with components related to the engagement of policymakers in research.^220^,^25^ These are the interactive model of researcher-policymaker collaboration,^26^ the Canadian Institutes of Health Research (CIHR) model of (IKT) and Knowledge to Action Cycle,^22^ EVIPNet Europe Situation Analysis Framework,^23^ the Conceptual framework for understanding and assessing research-policy partnerships,^20^ the Conceptual framework on Embedded Implementation Research^24 25^ and the SPIRIT Action Framework to assess policymakers’ research capacities and the engagement in and use of research.^2^ Additionally, we identified the following frameworks by scanning reference lists of relevant articles: the analytical framework for policy-relevant systematic reviews,^27^ the 7Ps Framework for Stakeholder Engagement (Patients and Public, Providers, Purchasers, Payers, Policymakers, Product makers, Principal investigators) and the Six Stages Model.^28^

### Documented enablers and barriers for policymaker engagement in research

Included documents reported 17 enablers and 14 barriers to the engagement of policymakers in research. We categorised the enablers and barriers as outlined in table 2. As groups, the individual level enablers and barriers were the most frequently mentioned in the literature (n=133), followed by organisational (n=92) and system/contextual level factors (n=29). However, as a single factor, the organisational level had the most frequently mentioned enabler and barrier.

**Table 2:** Summary of enablers and barriers for the engagement of policymakers in research processes, including number (n) of mentions of each factor

Level	Enabler (n)	Barrier (n)
Individual	Researchers Providing practical and expert advice to policymakers (14) Consistent, respectful and timely communication (7) Tailored communication strategies (ie, framing data in a clear, brief and direct manner) (6) Being useful to policymakers (6) Policymakers Intrinsic interest in research (5) Identifying the research needs to be addressed (3) Both Leveraging personal and professional networks (13) Mutual trust, respect and appreciation (10) Motivated leaders and champions (9) Continuous and iterative exchanges (6)	Researchers Lack of skills to understand policy-making processes and work in collaboration with policymakers (21) Limited and inconsistent engagement (4) Use overly complex language and lengthy reports (3) Lack of transparency in the research (2) Policymakers Limited time and resources for engaging in and conducting research while balancing other responsibilities (4) Lack or insufficient research skills (4) Both Mismatch in priorities, values, perceptions and expectations (13) Mistrust between researchers and policymakers (3)
Organisational	Institutionalising collaboration through partnerships, formal agreements and initiatives (ie, secondments, researcher in-residence) (18) Defining goals, roles, responsibilities and mechanisms for conflict resolution clearly from the outset of collaboration (16) Supportive institutions (11) Maintaining regular and open communication channels (7) Alignment with policy needs and priorities (5)	Lack of infrastructure, regulations, funding or communication channels (22) Differences in academic and policy needs, interests, objectives and incentives (7) Mismatch in timelines between researchers and policymakers, including funding timelines (6)
Contextual/system	Requirement of engagement by research funders (4) Integration of research into decision-making (4)	Health system organisation and structure (ie, centralised decision-making and bureaucratic processes) (10) Government turnover (9) Concentration of collaborative networks (2)

#### Enablers

The five most frequent enablers mentioned in the literature were (1) the institutionalisation of partnerships, initiatives and having formal agreements; (2) defining goals, roles, responsibilities and mechanisms of conflict resolution; (3) researchers providing practical and expert advice to policymakers; (4) leveraging personal and professional networks; and (5) having supportive institutions.

##### Individual level enablers

The most mentioned individual-level enabler is researchers being able and willing to provide objective, practical and expert advice to policymakers.^29^,^31^ This ‘behind the scenes’ support helps policymakers address pressing questions and relevant issues in their day-to-day work. Examples include evaluating claims made by other policymakers or stakeholders, helping contextualise evidence or answering questions that lack robust research evidence. ‘I would very frequently phone X [a researcher and clinician]….and say “X, I’ve just been told all this stuff, what do you think?”’ and he’d say, ‘Yeah, that sounds a bit right’ or ‘Be careful’^31^ highlighted one policymaker. Policymakers valued receiving expert advice on new policies, ideas or initiatives and used researchers as ‘sounding boards’.^30 31^ Being useful for policymakers was also noted as an enabler, with policymakers referring to researchers as ‘safety nets’ for justifying policy.^29 31^

Consistent, clear, respectful, and timely communication emerged as another relevant enabler for effective engagement.^2021 32^,^34^ Maintaining dialogue and resolving issues efficiently also supported engagement. Direct engagement included retreats, workshops and face-to-face meetings accompanied by written documents.^20 34^ Additionally, researchers were encouraged to inform policymakers throughout the research process.^32^ Tailoring communication strategies to the needs of policymakers was mentioned in several articles as an enabler,^2230 35^,^37^ with researchers needing to frame data clearly, briefly and directly. Policymakers emphasised that they want research findings that are actionable, user-friendly and practical.^30 36 37^

Regarding the individual-level enablers for policymakers, the intrinsic interest in research and identifying research needs were noted in the literature.^2530 38^,^40^ Policymakers with prior experience or interest in research were more likely to advance collaboration with researchers.^38^ This interest increased their receptivity to research and empowered policymakers to advocate for evidence-based practices within their organisations.^40^ For example, policymakers reported that collaborative initiatives like Policy BUDDIES helped them recognise the value of research evidence, creating demand for policy-relevant knowledge.^40^ Additionally, identifying specific problems or priorities for research encouraged policymakers to collaborate with researchers to improve their programmes.^25 38^

We found four enablers pertaining to both groups: leveraging personal and professional networks; having mutual trust, respect and appreciation; having motivated leaders and champions; and continuous and iterative engagements. Leveraging personal and professional networks was the most frequently mentioned enabler.^20 25 30 34 36 38 41^ Existing personal and institutional relationships and previous experiences facilitated collaboration.^41^ The willingness of policymakers to engage with researchers was often determined by the reputation and level of trust in individuals and their institutions.^30 36^ Alumni networks or colleague introductions initiated and maintained collaborative relationships.^30^ Motivated leaders and champions also initiated, sustained and expanded research collaboration.^2425 33 39^,^42^ They also contributed to a research-positive environment, driving ownership and collaboration. These leaders further promoted the value of research in policymaking and contributed to disseminating and implementing research findings.^24 40 42^

Mutual trust, respect and appreciation between policymakers and researchers emerged as an enabler in several contexts.^2022 25 30 34 40^,^43^ Continuous and iterative exchanges often strengthened this mutual trust and respect.^2540^,^42^ It also empowered policymakers to demand, appraise and use research.^40^ Understanding each other’s roles and constraints, as well as being appreciative of their context, structures, challenges and organisational environments, enabled collaboration and partnership.^20 30 43^ It also created space to negotiate and clarify research questions, solicit informal advice and adapt research to policymakers’ current needs.^29 40 42^

Understanding policymakers’ time constraints, Mendell *et al* highlighted the importance of carefully planning to ensure that time invested in engagement meaningfully contributed to research.^22^ Similarly, Varallyay *et al* noted that adjusting the intensity of involvement as needed for the research stage can make better use of policymakers’ limited availability.^25^

##### Organisational-level enablers

The most mentioned organisational-level enabler of policymaker engagement in research in the literature is institutionalising collaboration through partnerships, formal agreements and initiatives.^20 29 31 35 37 39–45^ These formalised structures, such as having Memoranda of Understanding (MoU), helped clarify roles and responsibilities while also advancing trust and accountability.^20 39^ Creating regular forums for dialogue and interaction facilitated finding the right person for collaboration.^43^ Having supportive institutions was another organisational enabler^29^,^3135 39 40 44^ that created environments that allow sustained interaction and stable partnerships. Additionally, some studies noted how institutions use incentives, for instance, by offering funding or paid time to engage with researchers, creating spaces for collaboration or allowing individuals to participate in workshops.^30 35 39 40 44^

Another organisational enabler involved defining and agreeing on the goals, roles, responsibilities and mechanisms for conflict resolution through documents like MoU, terms of reference or operating procedures.^20 21 25 29 31 42^ Defining these elements early in the process gave both parties clarity on how to manage and resolve conflicts.^20 25 42^ Nevertheless, Mirzoev *et al* noted that ‘actual roles and responsibilities of partners may be different to those in the formal document’, highlighting the need to monitor and adapt the implementation of formal agreements.^20^ Having clear goals, well-defined processes (including for publication), and shared risk (financial and reputational) were also important for partnerships.^29^ Alignment with policy needs and priorities was noted^29 38 39 45^ and considered necessary for establishing agreement and collaboration between policymakers and researchers.^29 45^ Finally, maintaining regular and open communication channels through forums, platforms and other spaces is another way institutions and organisations support researcher-policymaker engagement.^29 35 37 39 40 46^

##### Contextual/system-level enablers

At the system level, the two main enablers for policymaker engagement in research include the requirement of engagement by research funders^30 39^ and the integration of research into policy and decision-making processes.^25 29 38 47^ Funders, such as the CIHR, have promoted health system partnerships between researchers and policymakers.^31^ In practical terms, this engagement has often materialised in calls for proposals mandating the inclusion of policymakers as co-investigators. This approach aims to improve the research investments, as noted by a senior health official ‘Funders at every level want a better understanding on their investment in research and so, I think, the funding competitions have shifted in recent years, and are continuing to shift, to more application’.^31^ While policymakers recognise the value of these approaches, we found limited explicit acknowledgement in the literature regarding the time and resources necessary for continuous engagement. Additionally, the integration of research into decision-making processes through government-funded research centres can support collaborations, as seen in New South Wales.^29^ In the case of Nigeria, the creation of a presidential taskforce during the COVID-19 pandemic, which included academics, experts and policymakers, reduced ministerial division and eased decision-making.^47^

### Barriers

Regarding the barriers, the most frequently mentioned were (1) lack of regulations, infrastructure, funding or communication channels; (2) the lack of skills of researchers to understand policymaking processes and work in collaboration with policymakers; (3) the mismatch in priorities, values, perspectives and expectations; (4) government turnover; and (5) the health system organisation and structure.

#### Individual-level barriers

The most frequently mentioned individual-level barrier to policymaker engagement in research is that researchers do not understand policymaking processes and lack skills to collaborate with policymakers.^29 36 39 40 42 43^ This skill gap involved failing to appreciate the nuances of policy needs and having ‘naïve’ perceptions of the policy process and the policy environment, leading to miscommunication and missed opportunities for meaningful collaboration.^29 39 43^ For instance, one participant noted, ‘…they want records, like 30 years’ worth of records for a certain kind of condition, you know, that involves both our analytic people and health records, and that can’t be done for free’.^39^ Policymakers felt that researchers had a narrow approach and ignored the complexity of the problems they faced.^29 39^ The lack of preparation of researchers for working in the fast-paced policy environment was also noted by policymakers.^39^

Other individual-level barriers pertaining to researchers were related to communication between researchers and policymakers: limited and inconsistent engagement,^29 30 38 39^ providing overly complex language and lengthy reports^29 37^ and lack of transparency in the research.^20 30^ Some policymakers expressed concerns about the commitment of researchers who engaged little and inconsistently^30 38^ and who often failed to maintain communication after they obtained their research funding.^29 39^ Some studies highlighted that policymakers were disappointed when researchers failed to synthesise evidence or provide actionable recommendations.^29^ Failing to disclose important information affects engagement by leading to longer processes than anticipated.^30^

At the individual level, policymakers faced two main barriers to engaging in research and the research process. The first was limited time and resources while balancing other responsibilities.^2 29 38 39^ This resulted in minimal engagement as policymakers often have multiple competing priorities: ‘While ideally, they would like to co-produce research, in practice, they had not found this to be possible’.^29^ Another barrier was inadequate skills to understand research.^20 37 39 46^ Studies from South Africa and Zambia revealed that policymakers felt disempowered by their limited research skills, which restricted their participation in research.^20^

On the barriers pertaining to both groups, two key factors hinder the engagement: a mismatch in priorities, values, perceptions and expectations^29 30 38 39 43 48^,^30 36 40^ Although most discussions of the mismatch between the two groups referred to differences in the type of questions, timelines and approaches, a few studies also referred to policymakers not getting credit for their contributions or not being informed about research outputs.^29^ Additionally, mistrust was mentioned in a few studies in terms of researchers being seen as biased.^40^

#### Organisational-level barriers

The most commonly reported organisational-level barrier to policymaker engagement in research was the lack of infrastructure, regulations, funding and communication channels to support such engagement.^230^,^32 36 39 40 43 45 49^ For instance, Ministries of Health (MoHs) tend to lack formal mechanisms for individuals to engage in long-term engagement with research institutions, such as sabbaticals or secondments, with less than a third among 24 MoHs from different low- and middle-income countries (LMICs) having such arrangements.^49^ Without clear regulations and institutional frameworks for collaboration, engagement may be inconsistent and fragmented. Instead, it occurs irregularly based on individual interests and informal contacts. In addition, the absence of regulations can create uncertainty around roles and responsibilities, limiting accountability and continuity of collaboration. Budgetary constraints within organisations, understaffing of research-related positions, and a lack of organisational infrastructure to support partnerships were found to be barriers to engagements.^39 43^ Additionally, the complexity of administrative issues leads policymakers to prefer partnerships with specific institutions, such as previously contracted institutions, for which procurement processes are easier.^30 31 39^ The lack of formal forums and communication channels to ‘discuss issues and bring people together’ was also mentioned as a barrier.^43^

The other organisational-level barriers mentioned in the literature were differences in academic and policy needs, interests, objectives, and incentives^23 39 43^ and the mismatch in timelines^29 31 39 43^ between researchers and policymakers. Divergence in interests made collaboration harder to establish.^39^ Furthermore, the mismatch in timelines made research findings irrelevant to policymakers by the time they were available, with some policymakers feeling like research was a waste of time.^29^

#### Contextual/system-level barriers

The three barriers to policymaker engagement in research at the system level are health system organisation and structure, government turnover and the concentration of collaborative networks. Health system organisation, such as centralised decision-making, bureaucratic processes and communication challenges between different health system levels and external partners limited engagement.^20 33 37 39^ Frequent turnover among health managers and policymakers may disrupt continuity in partnerships and collaboration.^25 29 33 39 45^ For instance, new personnel may bring different priorities^45^ or delay work because relationships need to be re-established.^39^ Additionally, concentrating collaborative networks in a few selected organisations can endanger engagements when leadership changes.^39^

## Discussion

This scoping review aimed to identify and describe the literature on enablers and barriers to the engagement of policymakers in research, focusing on the perspectives of policymakers. It found that organisational factors, including institutionalising collaboration through partnerships; formal agreements and initiatives; and the lack of infrastructure, regulations, funding or communication channels, were the most frequently reported factors influencing the engagement of policymakers in research across different contexts. Other organisational factors, such as defining the goals of engagement, roles and responsibilities in collaboration, and mechanisms for conflict resolution, were frequently mentioned. Individual factors like researchers’ understanding of the policy-making processes and collaboration with policymakers, and being available to provide objective, practical and expert advice to policymakers were additional factors.

Many of the enablers and barriers we identified coincide with previous literature, including studies reporting the perspectives of researchers. For instance, several studies highlighted the importance of aligning research with policymakers’ timelines and needs,^50 51^ defining goals, roles and responsibilities for engagement,^6^ formalising collaboration through partnerships and agreements,^9^ maintaining regular communication,^26^ improving relationships and skills^51^ and providing research outputs that align with the needs of policymakers.^50 52^ In addition, various tools, checklists, and recommendations to improve this engagement already exist.^4 6 27 28^ Furthermore, the role of research funders in enabling collaboration is evident in the numerous initiatives that promote research-policy engagement activities.^4 9^ Building on this established knowledge, our study provides an up-to-date perspective on the factors that affect the engagement of policymakers in research, particularly in light of experiences during the COVID-19 pandemic and expansion of in-country capacity for evidence-based decision-making in many LMICs.

By focusing on the perspectives of policymakers, our study has identified some new ideas and strategies to improve engagement between researchers and policymakers in ways that improve research uptake and reduce waste. First, policymakers emphasised the importance of organisational factors rather than individual factors, highlighting the need for institutional support for both policymakers and researchers. We also identified potential ways of creating more equitable and sustainable engagement between researchers and policymakers that may not be apparent when considering researcher’s viewpoints alone. For instance, funders, research and decision-making institutions can create frameworks for engagement, including formal agreements, and provide resources for long-term organisational and individual collaboration. Future initiatives should consider and build on the existing evidence about the engagement initiatives between researchers and policymakers that have been effective^9^ and be designed to allow rigorous evaluation and continuous learning. In addition, the relatively few mentions of contextual/system-level factors compared with individual and organisational factors highlight the research gaps related to these factors. This level could benefit from further research.

Second, we found that concentrating collaborative networks in selected leaders or institutions can negatively affect the broader system by not allowing other policymakers and researchers to engage, develop their skills and establish partnerships. Funders, research and decision-making institutions can consider this when designing engagement initiatives, ensuring that collaboration and capacity-strengthening opportunities are distributed across a broader range of institutions. Furthermore, creating funding mechanisms and institutional policies that incentivise the expansion of collaborative networks can promote a decentralised and resilient ecosystem for engagement between researchers and policymakers. However, as policymakers have limited time to engage, funders, research and decision-making institutions should be mindful and avoid creating a ‘crowded’ space with competition between engagement initiatives. Rather, they can thoughtfully plan how new initiatives complement the current landscape.^9^

Third, although the enablers and barriers are typically presented as ‘independent’ factors, these occur within an ‘ecosystem’ where enablers and barriers interrelate and interact with each other to affect engagement by policymakers. For instance, factors such as institutionalised collaboration, policymakers with intrinsic interest in research, clear and timely communications between researchers and policymakers, and integration of research into policy and decision-making processes all characterise an environment where engagement is encouraged. However, finding which factors most significantly affect the engagement of policymakers in research and in which settings and contexts requires a systems thinking approach^53 54^ that is important when designing and evaluating new research-policy engagement initiatives. Policymaker engagement in research needs to evolve from ad-hoc, project-specific collaborations based on informal and individual relationships, to institutionalisation of co-production, where evidence use is integrated into decision-making practices. This progression can contribute to building knowledge systems that can learn and adapt.^55^

This scoping review captures the views of a diverse range of policymakers, from national and subnational health ministry officials and health programme managers to senior policymakers from ministries of finance, social development and other key sectors (online supplemental appendix F). While we did not specifically analyse the different perspectives by type or level of policymaker, their roles and responsibilities within their own institutions may also influence their engagement in the research process.

### Limitations

This study has identified a series of enablers and barriers to policymaker engagement in research from the policymakers’ perspective. Nevertheless, it faced a number of limitations related to its scope and available resources which have left open lines of research that merit further inquiry.

This review was narrower in scope than other systematic reviews, such as Oliver *et al*^51^ and Innvaer *et al*^52^, which incorporate more studies, but are focused on evidence use by policymakers. Our hypothesis that policymakers’ perspectives are of particular importance and less commonly addressed led us to focus on the narrower question of how policymakers view the enablers and barriers to research engagement. This explains the relatively small number of articles in our study. Furthermore, by focusing on studies that include the perspectives of policymakers who engaged in the research process, we acknowledge that studies we excluded, due to with limited or passive policymaker engagement, might have offered additional insights that our sample did not address.

Our review included high-, middle- and low-income countries, as well as experiences that were global, regional and local. However, we did not disaggregate our findings along these dimensions due to the small number of studies. Consequently, this study is limited in what it can say about differences in enablers and barriers across such contexts.

We also acknowledge that our selection criteria resulted in an uneven distribution of articles, especially regarding geographical representativity. To some degree, this is a natural consequence of finding few studies, which may reflect the limited number of policymaker engagement experiences, along with a low probability of having experiences documented in the public record. The articles may also exhibit publication bias, with authors and journals willing to report successful cases and reticent about reporting failed experiences. Limiting the sample to publications in English may also have excluded worthwhile experiences that might have been documented in other languages.

Although we relied on evidence in the literature regarding the benefits of engaging policymakers in research processes, we did not focus on this aspect. Thus, we have not judged the effectiveness or success of collaboration, coordination or partnership models. Likewise, we were more interested in finding the enablers and barriers for meaningful engagement than in identifying specific models for partnerships, coordination or collaboration. Thus, we acknowledge that the appropriate models to engage policymakers might differ depending on context and specific needs. Furthermore, we did not look at the differences in factors across contexts or specify where barriers to research engagement are more likely to occur. Future inquiries would be needed to establish the significance of context, particularly distinguishing low- and middle-income countries from high-income countries. Additionally, not all research is the same in terms of its utility for policy and how it engages policymakers.^56^ While recognising these differences is important, our study did not distinguish between research types and engagements, making this a consideration for future research.

Finally, we were unable to double-code studies due to limited resources. However, our process included all reviewers and was iterative, allowing us to review the included articles and coded segments several times when extracting relevant information and to capture and reflect content accurately. Being a descriptive scoping review, we did not make a critical appraisal of the evidence sources. However, we followed a pre-published protocol and rigorous methodology to limit bias in identifying themes and eliciting the perspectives of policymakers as presented in the included literature.^12 19^

## Conclusions

This scoping review explored the literature about the enablers and barriers to the engagement of policymakers in research, focusing on the perspectives of policymakers. Our study underscores the need for institutional support, widespread collaboration opportunities, and the interconnected nature of many factors within the research-policy ecosystem to offer insights derived from the recurring themes raised by policymakers. These can inform future initiatives to improve research-policy engagement and support evidence-based decision-making in health systems.

## Supplementary material

10.1136/bmjopen-2025-099720 online supplemental file 1
