# Supplementary material for: Enablers and barriers for policymaker engagement in health research from the perspective of policymakers: a scoping review
Source: BMJ Open. 2025 Aug 21;15(8):e099720. doi: 10.1136/bmjopen-2025-099720 (PMC12374644; doi:10.1136/bmjopen-2025-099720)
Supplement: online supplemental file 1 [file bmjopen-15-8-s001.docx]

**Supplementary file**

**Appendix A.** PRISMA-ScR checklist

**Preferred Reporting Items for Systematic reviews and Meta-Analyses extension for Scoping Reviews (PRISMA-ScR) Checklist**

| **SECTION** | **ITEM** | **PRISMA-ScR CHECKLIST ITEM** | **REPORTED ON PAGE #** |
| --- | --- | --- | --- |
| **TITLE** | | | |
| Title | 1 | Identify the report as a scoping review. | 1 |
| **ABSTRACT** | | | |
| Structured summary | 2 | Provide a structured summary that includes (as applicable): background, objectives, eligibility criteria, sources of evidence, charting methods, results, and conclusions that relate to the review questions and objectives. | 2 |
| **INTRODUCTION** | | | |
| Rationale | 3 | Describe the rationale for the review in the context of what is already known. Explain why the review questions/objectives lend themselves to a scoping review approach. | 4 |
| Objectives | 4 | Provide an explicit statement of the questions and objectives being addressed with reference to their key elements (e.g., population or participants, concepts, and context) or other relevant key elements used to conceptualize the review questions and/or objectives. | 4 |
| **METHODS** | | | |
| Protocol and registration | 5 | Indicate whether a review protocol exists; state if and where it can be accessed (e.g., a Web address); and if available, provide registration information, including the registration number. | 4 |
| Eligibility criteria | 6 | Specify characteristics of the sources of evidence used as eligibility criteria (e.g., years considered, language, and publication status), and provide a rationale. | 4-5 |
| Information sources* | 7 | Describe all information sources in the search (e.g., databases with dates of coverage and contact with authors to identify additional sources), as well as the date the most recent search was executed. | 5 |
| Search | 8 | Present the full electronic search strategy for at least 1 database, including any limits used, such that it could be repeated. | Supplementary appendix p.4-6 |
| Selection of sources of evidence† | 9 | State the process for selecting sources of evidence (i.e., screening and eligibility) included in the scoping review. | 5-6 |
| Data charting process‡ | 10 | Describe the methods of charting data from the included sources of evidence (e.g., calibrated forms or forms that have been tested by the team before their use, and whether data charting was done independently or in duplicate) and any processes for obtaining and confirming data from investigators. | 6 |
| Data items | 11 | List and define all variables for which data were sought and any assumptions and simplifications made. | 6 |
| Critical appraisal of individual sources of evidence§ | 12 | If done, provide a rationale for conducting a critical appraisal of included sources of evidence; describe the methods used and how this information was used in any data synthesis (if appropriate). | NA |
| Synthesis of results | 13 | Describe the methods of handling and summarizing the data that were charted. | 6 |
| **RESULTS** | | | |
| Selection of sources of evidence | 14 | Give numbers of sources of evidence screened, assessed for eligibility, and included in the review, with reasons for exclusions at each stage, ideally using a flow diagram. | 6-7 |
| Characteristics of sources of evidence | 15 | For each source of evidence, present characteristics for which data were charted and provide the citations. | 7 |
| Critical appraisal within sources of evidence | 16 | If done, present data on critical appraisal of included sources of evidence (see item 12). | NA |
| Results of individual sources of evidence | 17 | For each included source of evidence, present the relevant data that were charted that relate to the review questions and objectives. | 7-16 |
| Synthesis of results | 18 | Summarize and/or present the charting results as they relate to the review questions and objectives. | 7-16 |
| **DISCUSSION** | | | |
| Summary of evidence | 19 | Summarize the main results (including an overview of concepts, themes, and types of evidence available), link to the review questions and objectives, and consider the relevance to key groups. | 16 |
| Limitations | 20 | Discuss the limitations of the scoping review process. | 17-18 |
| Conclusions | 21 | Provide a general interpretation of the results with respect to the review questions and objectives, as well as potential implications and/or next steps. | 18 |
| **FUNDING** | | | |
| Funding | 22 | Describe sources of funding for the included sources of evidence, as well as sources of funding for the scoping review. Describe the role of the funders of the scoping review. | 19 |

JBI = Joanna Briggs Institute; PRISMA-ScR = Preferred Reporting Items for Systematic reviews and Meta-Analyses extension for Scoping Reviews.

* Where *sources of evidence* (see second footnote) are compiled from, such as bibliographic databases, social media platforms, and Web sites.

† A more inclusive/heterogeneous term used to account for the different types of evidence or data sources (e.g., quantitative and/or qualitative research, expert opinion, and policy documents) that may be eligible in a scoping review as opposed to only studies. This is not to be confused with *information sources* (see first footnote).

‡ The frameworks by Arksey and O’Malley (6) and Levac and colleagues (7) and the JBI guidance (4, 5) refer to the process of data extraction in a scoping review as data charting*.*

§ The process of systematically examining research evidence to assess its validity, results, and relevance before using it to inform a decision. This term is used for items 12 and 19 instead of "risk of bias" (which is more applicable to systematic reviews of interventions) to include and acknowledge the various sources of evidence that may be used in a scoping review (e.g., quantitative and/or qualitative research, expert opinion, and policy document).

*From:* Tricco AC, Lillie E, Zarin W, O'Brien KK, Colquhoun H, Levac D, et al. PRISMA Extension for Scoping Reviews (PRISMAScR): Checklist and Explanation. Ann Intern Med. 2018;169:467–473. [doi: 10.7326/M18-0850](http://annals.org/aim/fullarticle/2700389/prisma-extension-scoping-reviews-prisma-scr-checklist-explanation).**Appendix B.** Original protocol

**Scoping Review Protocol**

**Title**

Enablers and barriers for policy-maker engagement in research agenda-setting: a scoping review

**Introduction**

Including knowledge end-users in the research process reduces research waste through a co-production model, instead of tokenistic engagement (1). Co-production of research can improve the quality and relevance of research, increase its effective utilization, increase accountability and transparency to research funders, and empower and give ownership to policy-makers (2,3). Knowledge users can be involved at various levels: leading (making key decisions), controlling (developing the inclusion criteria), influencing (assisting with data extraction), contributing (helping in the prioritization of research priorities) and receiving the results of the research. However, co-production can also increase the time to complete research, and the financial resources needed required. In general, when feasible and appropriate, knowledge users should be included from the beginning of the research (4).

Research-policy partnerships are a well-established means for advancing evidence-informed policy (5). However, policymaking is a complex and non-linear process influenced by the context, ideologies, and values of policy-makers, where evidence is one of many inputs. Moreover, the process is country-specific and depends on policy structures and mechanisms (5–7).

By applying effective and efficient policy-maker engagement approaches, evidence-based organizations can optimize their resources and capacities and achieve meaningful impact. This scoping review proposes to understand current frameworks and evidence on policy-maker engagement to 1) align research agendas with the needs of policy-makers, 2) allow policy-makers to choose, design and execute agendas and policies, 3) improve the implementation of day-to-day policies, and 4) guide policy-makers to evaluate the results from the decisions made(8). A preliminary search for existing systematic and scoping reviews on the topic has been conducted in the Cochrane Database of Systematic Review and JBI Evidence Synthesis, finding no existing reviews on this topic.

**Objectives**

The objective of this scoping review is to understand current frameworks and evidence on policy-maker engagement. The specific objectives are to:

- Summarize the enablers and barriers for policy-maker engagement across key processes of research and decision-making cycles
- Map actionable opportunities to enable policy-makers to engage in research across key phases of research decision-making, production, and use.

The specific research questions (RQ) for our scoping review are:

- RQ1. What are the enablers and barriers in aligning research agendas with the needs of policy-makers?
- RQ2. What are the enablers and barriers in accessing the evidence needed by policy-makers to choose, design and execute strategies?
- RQ3. What are the enablers and barriers for policy-makers to improving implementation day-to-day?
- RQ4. What are the enablers and barriers of policy-makers to evaluate the results from the decisions made?

**Inclusion criteria and exclusion criteria**

| **Inclusion criteria** | **Exclusion criteria** |
| --- | --- |
| **Population (Participants)** | |
| Must include policy-makers, decision-makers or regulators at any level (national or sub-national).  Policy-makers, decision-makers and regulators are defined as “individuals and organizations that create, monitor and oversee policies or regulations of healthcare-related issues, such as federal, state and local government agencies, medical and professional organizations and clinical guidelines developers” (9). | Exclude if it does not include policy-makers or decision-makers. |
| **Interventions** | |
| Policy-makers or decision-makers are actively engaged in any stage of the research process.  Engagement was defined as “an iterative process of actively soliciting the knowledge, experience, judgement and values of individuals selected to represent a broad range of direct interests in a particular issue, for the dual purposes of: creating a shared understanding [and] making relevant, transparent and effective decisions” (9).  Potential points of engagement:   - Topic selection (priority-setting) - Conceptualize and design (develop question or protocol) - Search and data collection (locate literature, collect and appraise evidence) - Data synthesis (data analysis, data interpretation) - Knowledge product (manuscript/report, briefs) - Uptake and evaluation (monitor use and impact)   Methods of engagement:   - In-person/telephone meetings - Email communications - Document sharing and feedback - Surveys, focus groups, interviews - Workshops, webinars, educational rounds - Nominal group techniques, Delphi | Policy-makers or decision-makers were only passively engaged:   - Presented with research findings (at conferences, manuscript publication) - Given research findings without opportunities for feedback |
| **Comparators** | |
| Articles can include or not a comparator group. | Not applicable. |
| **Outcomes** | |
| Must report factors that may help or hinder policy-makers or decision-makers to engage in the research process and decision-making cycles. | No relevant information provided. |
| **Study design** | |
| Any study design including:   - Experimental studies - Quasi-experimental studies - Observational studies - Systematic reviews and meta-analysis | Editorials, commentaries. |
| **Setting** | |
| All settings are eligible for inclusion. |  |

**Methods**

The scoping review will follow the Joanna Briggs Institute Methods Manual for scoping reviews (10). The scoping review methodology is useful when exploring a diverse knowledge base, as is the case (10). The findings will be reported following the Preferred Reporting Items for Systematic reviews and Meta-Analyses extension for Scoping Reviews (PRISMA-ScR) Checklist (11). Full protocol will be registered in the Open Science Framework (<https://osf.io/>).

*Search strategy*

The first step of the literature search will include the following electronic databases: MEDLINE, Cochrane Library, Social Policy and Practice, Campbell Collaboration, Health Systems Evidence and the World Bank e-Library. These databases will be supplemented by searching Grey literature sources (i.e., Google Scholar, WHO Global Index Medicus and VHL Regional Portal). Multiple search strategies will be developed for this review, reflecting the different approaches to identify this type of evidence. Additional sources will be searched through identified reports and articles, and the Alliance articles. The articles will be restricted to those in English, published from 2007 onwards, as this start date coincides with the publication of the “Sound Choices” report (6). The search strategies are detailed in Annex A.

*Study selection*

Title and abstract screening and full-text screening will be performed by two reviewers, independently. Full-text will be assessed for relevance to the four research questions. Any disagreements will be resolved by the decision of a third reviewer. A flowchart of review process will detail the search, source selection, duplicates, full-text retrieval, additional search, data extraction and presentation of the evidence. We will use specialized software for the management of the results of the research. We will conduct a pilot testing of source selectors following the JBI framework (10).

*Data extraction*

We identified three frameworks to guide our data collection (Annex B): the SPIRIT Action Framework, the Evidence needs of health system decision-makers and the conceptual framework of evidence-informed health policymaking (6,8,14). These frameworks emphasize different components or aspects of the policy process, and its influencers. Data will be extracted and collected in an excel spreadsheet. A pilot step will be performed by two members of the review team. As the policy process is a complex process, we propose a “complementary” use of frameworks to identify key components of enablers and barriers to policy-maker engagement in the existing literature. The combination of frameworks aims to explain empirical data using insights from multiple frameworks. This approach has the advantage of removing assumptions that one framework is better than another and enables greater understanding of the topic (12,13). Once results are extracted from the included sources, we will map them.

*Data presentation and analysis*

Initially, the three frameworks will inform our analysis. As the process will be iterative, other relevant frameworks might be added. We propose to perform a qualitative content analysis, with basic coding of data using NVivo. We will present search results in a PRISMA flow diagram (with information of the identification, screening, eligibility and included articles). A descriptive analysis of the included articles will be summarized in a table including the year of publication, geographic region, article type and funding source type. We will summarize the enablers and barriers in a table containing year of publication, country, point of engagement and method(s) of engagement.

**References**

1. Pollock D, Alexander L, Munn Z, Peters MDJ, Khalil H, Godfrey CM, et al. Moving from consultation to co-creation with knowledge users in scoping reviews: Guidance from the JBI Scoping Review Methodology Group. JBI Evid Synth. 2022 Apr 1;20(4):969–79.

2. Oliver K, Kothari A, Mays N. The dark side of coproduction: Do the costs outweigh the benefits for health research? Vol. 17, Health Research Policy and Systems. BioMed Central Ltd.; 2019.

3. Loncarevic N, Andersen PT, Leppin A, Bertram M. Policymakers’ research capacities, engagement, and use of research in public health policymaking. Int J Environ Res Public Health. 2021 Nov 1;18(21).

4. Chalmers I, Bracken MB, Djulbegovic B, Garattini S, Grant J, Gülmezoglu AM, et al. How to increase value and reduce waste when research priorities are set. The Lancet [Internet]. 2014 Jan;383(9912):156–65. Available from: https://linkinghub.elsevier.com/retrieve/pii/S0140673613622291

5. Banks E, Haynes A, Lovett R, Yadav UN, Agostino J. Output-orientated policy engagement: a model for advancing the use of epidemiological evidence in health policy. Vol. 21, Health Research Policy and Systems. BioMed Central Ltd; 2023.

6. Alliance for Health Policy and Systems Research Sound Choices Enhancing Capacity for Evidence-Informed Health Policy.

7. Loncarevic N, Andersen PT, Leppin A, Bertram M. Policymakers’ research capacities, engagement, and use of research in public health policymaking. Int J Environ Res Public Health. 2021 Nov 1;18(21).

8. Sheikh K, Kwamie A, Ghaffar A. Using Health Research for Evidence-Informed Decisions in Health Systems in LMICs. In: Making Health Systems Work in Low and Middle Income Countries [Internet]. Cambridge University Press; 2022. p. 354–67. Available from: https://www.cambridge.org/core/product/identifier/9781009211086%23CN-bp-23/type/book_part

9. Deverka PA, Lavallee DC, Desai PJ, Esmail LC, Ramsey SD, Veenstra DL, et al. Stakeholder participation in comparative effectiveness research: defining a framework for effective engagement. J Comp Eff Res [Internet]. 2012 Mar;1(2):181–94. Available from: https://becarispublishing.com/doi/10.2217/cer.12.7

10. Peters MD, Godfrey C, McInerney P, Munn Z, Tricco AC, Khalil H. Chapter 11: Scoping reviews. In: JBI Manual for Evidence Synthesis [Internet]. JBI; 2020. Available from: https://jbi-global-wiki.refined.site/space/MANUAL/4687342/Chapter+11%3A+Scoping+reviews

11. Tricco AC, Lillie E, Zarin W, O’Brien KK, Colquhoun H, Levac D, et al. PRISMA extension for scoping reviews (PRISMA-ScR): Checklist and explanation. Vol. 169, Annals of Internal Medicine. American College of Physicians; 2018. p. 467–73.

12. Cairney P. Standing on the Shoulders of Giants: How Do We Combine the Insights of Multiple Theories in Public Policy Studies? Policy Studies Journal. 2013 Feb;41(1):1–21.

13. Townsend B, Strazdins L, Harris P, Baum F, Friel S. Bringing in critical frameworks to investigate agenda-setting for the social determinants of health: Lessons from a multiple framework analysis. Soc Sci Med. 2020 Apr 1;250.

14. Redman S, Turner T, Davies H, Williamson A, Haynes A, Brennan S, et al. The SPIRIT Action Framework: A structured approach to selecting and testing strategies to increase the use of research in policy. Soc Sci Med [Internet]. 2015 Jul 1;136–137:147–55. Available from: https://linkinghub.elsevier.com/retrieve/pii/S0277953615002828

*Annex A: Search Strategy*

Main databases

| **Database** | **Limits** | **Searching Strategy** |
| --- | --- | --- |
| MEDLINE | 2007 to current (25 April 2023)  English | Engag*.mp. [mp=title, abstract, heading word, drug trade name, original title, device manufacturer, drug manufacturer, device trade name, keyword heading word, floating subheading word, candidate term word]  AND  (Polic* or decision*).mp. [mp=title, book title, abstract, original title, name of substance word, subject heading word, floating sub-heading word, keyword heading word, organism supplementary concept word, protocol supplementary concept word, rare disease supplementary concept word, unique identifier, synonyms]  AND  Evidence-inform*.mp. [mp=title, book title, abstract, original title, name of substance word, subject heading word, floating sub-heading word, keyword heading word, organism supplementary concept word, protocol supplementary concept word, rare disease supplementary concept word, unique identifier, synonyms] |
| Cochrane Library | January 2007 to April 2023 | (Engag*):ti,ab,kw AND (polic*):ti,ab,kw OR (decision*):ti,ab,kw AND (evidence-inform*):ti,ab,kw with Cochrane Library publication date Between Jan 2007 and Apr 2023, in Cochrane Reviews, Cochrane Protocols, Trials, Clinical Answers, Special Collections (Word variations have been searched). |
| Social Policy and Practice | 2007 to current | Engag*.mp. [mp=abstract, title, publication type, heading word, accession number]  AND  Polic*.mp. [mp=abstract, title, publication type, heading word, accession number] OR Decision*.mp. [mp=abstract, title, publication type, heading word, accession number]  AND  Evidenc*.mp. [mp=abstract, title, publication type, heading word, accession number] OR evidence-inform*.mp. [mp=abstract, title, publication type, heading word, accession number] |
| Campbell Collaboration | 1 January 2007 to 26 April 2023  English | Keyword: Engagement" OR "Policy" OR "Decision" OR "Evidence-informed"  Coordinating group(s): Business and Management, International Development, Knowledge Translation and Implementation, Methods and Social Welfare. Coordinating group(s): Business and Management, International Development, Knowledge Translation and Implementation, Methods and Social Welfare. |
| Health Systems Evidence | 2007 to 2023 | ((Engage OR Engagement OR engaging) AND (Decision OR Policy) AND (evidence)) |
| World Bank e-Library | January 2007 to April 2023  Journal Articles | “Engage” AND “decision OR policy” AND “evidence-informed” |

Supplementary databases

| Google Scholar | 2007 to 2023  In the title of the article  English | *(allintitle: Policy engagement Policy OR Decisionmaker OR policymaker OR "policy maker" OR "policy maker" OR "decision maker" OR engagement OR evidence OR "evidence informed" -patient -caregiver -community)* |
| --- | --- | --- |
| WHO Global Index Medicus | 2007 to 2023  Abstract  English | (ab:policy OR ab:decision) AND (ab:engage OR ab:engaging OR ab:engagement)  Search detail: ab:((ab:policy OR ab:decision) AND (ab:engage OR ab:engaging OR ab:engagement)) AND ( la:("en")) AND (year_cluster:[2007 TO 2023]) |
| VHL Regional Portal | 2007 to 2023  Abstract  Main subject (health policy, policy making, politics, global health, health priorities, public policy, program evaluation, national health programs, health planning)  English | (ab:policy OR ab:decision) AND (ab:engage OR ab:engaging OR ab:engagement)  Search detail: (ab:(policy)) OR (ab:(decision)) AND (ab:(engage)) OR (ab:(engagement)) OR (ab:(engaging)) AND NOT (ab:(patient)) AND NOT (ab:(community)) AND NOT (ab:(caregiver)) AND ( mj:("Health Policy" OR "Policy Making" OR "Politics" OR "Global Health" OR "Health Priorities" OR "Public Policy" OR "Program Evaluation" OR "National Health Programs" OR "Health Planning") AND la:("en") AND type:("article")) AND (year_cluster:[2007 TO 2023]) |

*Annex B: Frameworks*

1. SPIRIT Action Framework

| 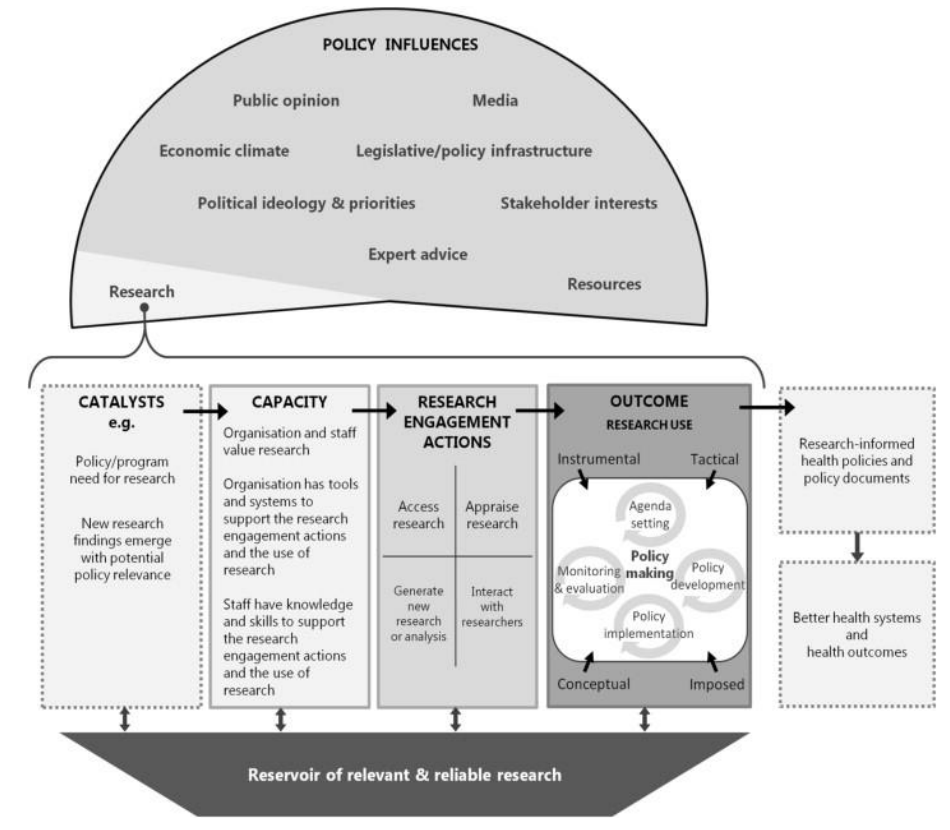 |
| --- |

1. Evidence needs of health system decision-makers

| 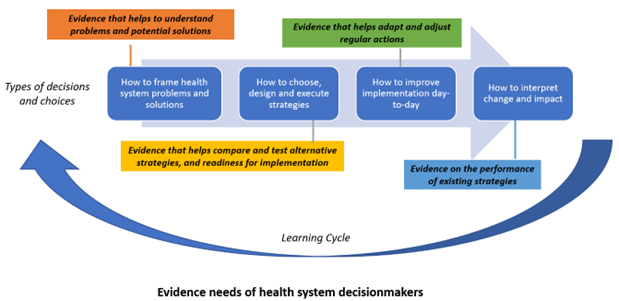 |
| --- |

1. Conceptual framework of evidence-informed health policymaking

| 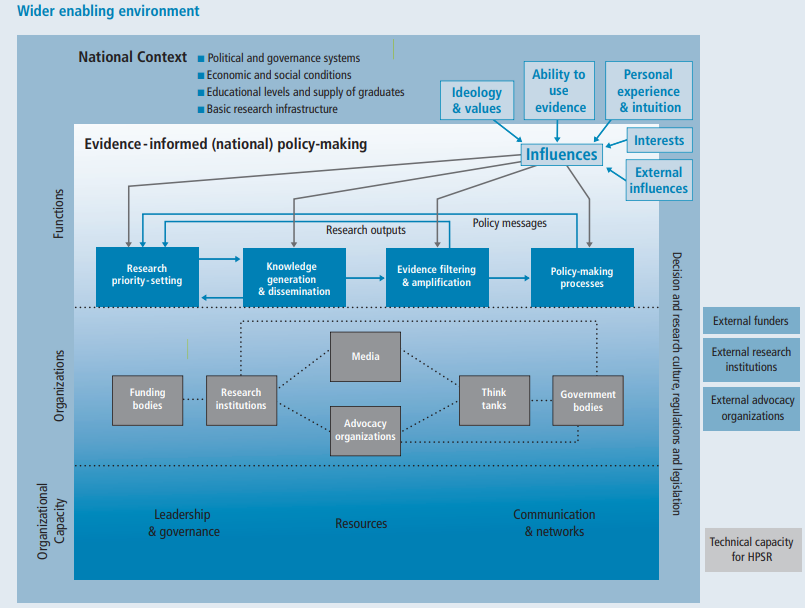 |
| --- |

Framework references:

Redman S, Turner T, Davies H, Williamson A, Haynes A, Brennan S, et al. The SPIRIT Action Framework: A structured approach to selecting and testing strategies to increase the use of research in policy. Soc Sci Med [Internet]. 2015 Jul 1;136–137:147–55. Available from: <https://linkinghub.elsevier.com/retrieve/pii/S0277953615002828>

Sheikh K, Kwamie A, Ghaffar A. Using Health Research for Evidence-Informed Decisions in Health Systems in LMICs. In: Making Health Systems Work in Low and Middle Income Countries [Internet]. Cambridge University Press; 2022. p. 354–67. Available from: <https://www.cambridge.org/core/product/identifier/9781009211086%23CN-bp-23/type/book_part>

Sound Choices. Enhancing capacity for evidence-informed health policy, Alliance HPSR, WHO, Geneva, Switzerland, ISBN 978-92-4-159590-2, 168 pp.

**Appendix C:** Search Strategy and Results

Main databases

| **Database** | **Limits** | **Searching Strategy** | **Total Records** | **Records after duplicate removal** | **Records after title and abstract screening** |
| --- | --- | --- | --- | --- | --- |
| MEDLINE | 2007 to current (25 April 2023)  English | Engag*.mp. [mp=title, abstract, heading word, drug trade name, original title, device manufacturer, drug manufacturer, device trade name, keyword heading word, floating subheading word, candidate term word]  AND  (Polic* or decision*).mp. [mp=title, book title, abstract, original title, name of substance word, subject heading word, floating sub-heading word, keyword heading word, organism supplementary concept word, protocol supplementary concept word, rare disease supplementary concept word, unique identifier, synonyms]  AND  Evidence-inform*.mp. [mp=title, book title, abstract, original title, name of substance word, subject heading word, floating sub-heading word, keyword heading word, organism supplementary concept word, protocol supplementary concept word, rare disease supplementary concept word, unique identifier, synonyms] | 384 | 370 | 22 |
| Cochrane Library | January 2007 to April 2023  English | (Engag*):ti,ab,kw AND (polic*):ti,ab,kw OR (decision*):ti,ab,kw AND (evidence-inform*):ti,ab,kw with Cochrane Library publication date Between Jan 2007 and Apr 2023, in Cochrane Reviews, Cochrane Protocols, Trials, Clinical Answers, Special Collections (Word variations have been searched). | 33 | 30 | 0 |
| Social Policy and Practice | 2007 to current (05/05/23)  English | Engag*.mp. [mp=abstract, title, publication type, heading word, accession number]  AND  Polic*.mp. [mp=abstract, title, publication type, heading word, accession number] OR Decision*.mp. [mp=abstract, title, publication type, heading word, accession number]  AND  Evidenc*.mp. [mp=abstract, title, publication type, heading word, accession number] OR evidence-inform*.mp. [mp=abstract, title, publication type, heading word, accession number] | 1064 | 909 | 3 |
| Campbell Collaboration | 1 January 2007 to 26 April 2023  English | "Engagement" OR "Policy" OR "Decision" OR "Evidence-informed"  Coordinating group(s): Business and Management, International Development, Knowledge Translation and Implementation, Methods and Social Welfare. | 37 | 36 | 0 |
| Health Systems Evidence | 2007 to 2023 (26/04/23) | ((Engage OR Engagement OR engaging) AND (Decision OR Policy) AND (evidence)) | 95 | 89 | 1 |
| World Bank e-Library | January 2007 to April 2023  Journal Articles | “Engage” AND “decision OR policy” AND “evidence-informed” | 349 | 349 | 0 |

Supplementary databases

| **Database** | **Limits** | **Searching Strategy** | **Total Records** | **Records after duplicate removal** | **Records after title and abstract screening** |
| --- | --- | --- | --- | --- | --- |
| Google Scholar | 2007 to 2023 (07/06/23)  In the title of the article  English | *(allintitle: Policy engagement Policy OR Decisionmaker OR policymaker OR "policy maker" OR "policy maker" OR "decision maker" OR engagement OR evidence OR "evidence informed" -patient -caregiver -community)* | 2330 (only 998 retrieved, most relevant) | 975 | 4 |
| WHO Global Index Medicus | 2007 to 2023 (07/06/23)  Abstract  English | (ab:policy OR ab:decision) AND (ab:engage OR ab:engaging OR ab:engagement)  Search detail: ab:((ab:policy OR ab:decision) AND (ab:engage OR ab:engaging OR ab:engagement)) AND ( la:("en")) AND (year_cluster:[2007 TO 2023]) | 142 | 136 | 1 |
| VHL Regional Portal | 2007 to 2023 (07/06/23)  Abstract  Main subject (health policy, policy making, politics, global health, health priorities, public policy, program evaluation, national health programs, health planning)  English | (ab:(policy)) OR (ab:(decision)) AND (ab:(engage)) OR (ab:(engagement)) OR (ab:(engaging)) AND NOT (ab:(patient)) AND NOT (ab:(community)) AND NOT (ab:(caregiver)) AND ( mj:("Health Policy" OR "Policy Making" OR "Politics" OR "Global Health" OR "Health Priorities" OR "Public Policy" OR "Program Evaluation" OR "National Health Programs" OR "Health Planning") AND la:("en") AND type:("article")) AND (year_cluster:[2007 TO 2023]) | 2552 | 2490 | 28 |

**Appendix D:** Codebook

|  | **Parent code** | **Sub-code** | **Description** | **Category** |
| --- | --- | --- | --- | --- |
| Characteristics of the initiative |  |  | This code applies to the situational background in which policymaker research engagement occurs. It includes aspects like the geographical context, the characteristics of those involved, including their capacities, and how the policymakers are engaged. |  |
|  | Actors’ characteristics |  | This code includes the attributes and qualities of key stakeholders involved in the engagement process. Characteristics may refer to their expertise, experience, roles, and motivations for engagement. |  |
|  |  | Researchers and research institutions | This code refers to individuals and organizations involved in conducting scientific research. |  |
|  |  | Policymakers and government | This code refers to individuals and organizations involved in the research process. |  |
|  | Catalyst |  | The code refers to events, conditions, or factors triggering the initiation or acceleration of research activities between policymakers and researchers. This can include recognizing a specific need for research to address gaps in knowledge, or new funding opportunities that enable the undertaking of research projects. |  |
|  |  | Need for research | Describes the driving factors behind the initiation of engagement, including identified gaps in knowledge, demand for evidence to inform policy, or emerging health and social issues. |  |
|  |  | New funding | Relates to the availability of new financial resources or funding opportunities that enable research and policymaker engagement. |  |
|  | Capacity |  | Focuses on the capabilities and resources available to policymakers for engaging with researchers distinguishing between institutional (organizational level) and individual (personal skills and knowledge) capacities. |  |
|  |  | Institutional | Institutional capacity refers to the ability of government institutions and organizations to carry out research activities effectively. It encompasses infrastructure, funding, human resources, operational processes, and governance structures that enable (or not) the use of research in decision-making. |  |
|  |  | Individual | Individual capacity highlights the skills, knowledge, and competencies of policymakers involved in the research process. It can also include opportunities and challenges to build or maintain research capacities. |  |
|  | Type of engagement in research |  | This code refers to the processes and methods through which policymakers are involved in the research process. This engagement can occur at different points (e.g., design, implementation, dissemination) and employ various methods (e.g., participatory action research, consultations). |  |
|  |  | Point of Engagement | Identifies the stage(s) in the research process where policymaker engagement occurs, from agenda-setting to evaluation. |  |
|  |  | Method of engagement | Details the approaches and techniques used to involve policymakers in research, such as surveys, workshops, participatory methods, advisory committees, etc. |  |
|  | Outcome (Research use and impact) |  | This code refers to **the impact** of policymaker engagement on research utilization by decision-makers. It reflects how the engagement led to different policy outcomes. |  |
|  |  | Agenda Setting | This code relates to the impact of identifying and prioritizing issues to be addressed within the policy framework. |  |
|  |  | Policy development | This code refers to the impact of formulating policies based on research evidence. |  |
|  |  | Policy Implementation | This code is related to the impact of the execution of policies, including the allocation of resources. |  |
|  |  | Monitoring and evaluation | This code concerns policies' impact, their effectiveness in being implemented as planned, and how they achieve the expected outcomes. |  |
|  | Knowledge |  | The code refers to the knowledge gained or needed by policymakers involved in the engagement process. |  |
|  |  | Learning | The process of adjusting strategies or behaviours based on new learning or feedback. |  |
|  |  | Gaps | The knowledge or skill gaps in policymakers or researchers that affect the engagement. |  |
|  | **Parent code** | **Sub-code** | **Description** | **Category** |
| Factors of the engagement |  |  |  |  |
|  | Alignment |  | This code relates to how researchers' and policymakers’ interests, needs, and priorities converge or diverge, affecting collaboration and outcomes. |  |
|  |  | Needs and priorities | The alignment (or misalignment) of research and researcher activities with the activities, needs, interests or priorities of policymakers. | Enabler  Barrier |
|  |  | Motivation and incentives | The alignment (or misalignment) of motivation or incentives for the engagement and/or collaboration between policymakers and researchers. | Enabler  Barrier |
|  | Communication |  | Communication includes the strategies, methods, and channels used to share information and insights during their engagement with researchers. |  |
|  |  | Channels/mode of communication | This code describes how the methods and platforms used to communicate among stakeholders contributed to the engagement. | Enabler  Barrier |
|  |  | Frequency of communication | This code refers to how often stakeholders communicate, influencing relationship building, trust, and project coordination. | Enabler  Barrier |
|  |  | Characteristics of the communication | This code describes the characteristics of the interaction and information sharing between researchers and policymakers (i.e. clear, short, direct language). | Enabler  Barrier |
|  | Trust |  | This code refers to the level of confidence and reliability among researchers and policymakers. |  |
|  |  | Establishment | The code refers to building initial trust, which could be related to transparency, communication, and demonstrated reliability. | Enabler  Barrier |
|  |  | Maintenance | This code refers to the actions that sustain trust between researchers and policymakers. This could be achieved through commitment and open communication. | Enabler  Barrier |
|  |  | Breach and recovery | The code refers to instances where trust is compromised and the efforts made to rebuild it. | Enabler  Barrier |
|  | Collaboration |  | This code refers to the processes and situations leading (or hindering) the formation and preservation of partnerships between policymakers and researchers at the individual or organizational level. It also includes how policymakers and researchers solve or address conflicts. |  |
|  |  | Formation | The processes and situations leading (or hindering) to establishing collaboration between policymakers and researchers. | Enabler  Barrier |
|  |  | Partnership | The processes and situations leading (or hindering) to cooperation through formal or informal agreements include roles, responsibilities, and expectations. | Enabler  Barrier |
|  |  | Dynamics | Mechanisms and situations arise during collaboration, as well as strategies for addressing disagreements or conflicts, including how consensus is achieved. | Enabler  Barrier |

**Appendix E.** Schematic representation of coding structure

**Appendix F.** List of included papers, title, objective and characteristics of policymakers or decision-makers involved in the study

| **No** | **Author, year** | **Title** | **Main study objective** | **Characteristics of policymakers or decision-makers involved** |
| --- | --- | --- | --- | --- |
| 1 | Abubakar I, 2021 | Lessons from co-production of evidence and policy in Nigeria's COVID-19 response. | To describe a co-production model in Nigeria during COVID-19. | Political decision-makers and health policymakers at the national and state levels, government agencies, and key members of the PTF on COVID-19 (ministries of health, finance, disaster management, aviation, and foreign affairs). |
| 2 | Badakhshan A, 2018 | Priority-setting in health research in Iran: a qualitative study on barriers and facilitators. | To assess the barriers and facilitators of priority-setting exercises/measures in Iran. | Health researchers working as healthcare or health research managers at different levels of the health system with experience in research management and being engaged in health research priority-setting. This included individuals working at the Ministry of Health and Medical Education, National Institute of Health Research, and the National Institute for Medical Research Development. |
| 3 | Bowen S, 2019 | Experience of Health Leadership in Partnering With University-Based Researchers in Canada - A Call to "Re-imagine" Research | To explore the experience and perspectives of senior health managers in health service organizations with health organization-university research partnerships. | Senior health managers in health service organizations across Canada who had an active leadership role in research partnerships and held support and coordination functions. |
| 4 | Cambe MI, 2022 | The Use of Research for Health Systems Policy Development and Implementation in Mozambique: A Descriptive Study. | To explore the relationship between health policy and systems research (HPSR) and health policy development and implementation in Mozambique. | Policymakers involved in national and subnational health policy decisions. |
| 5 | Ellen ME, 2018 | How is the use of research evidence in health policy perceived? A comparison between the reporting of researchers and policymakers. | To compare the perceptions of Israeli health systems and policy researchers and health services policymakers regarding the role of HPSR, factors influencing its uses and potential facilitators and barriers to HPSR, and implementation of KT and exchange activities. | Health policymakers directly involved in health policy-making, including officials from the Knesset, Israel’s MOH, the Ministry of Finance, health services organizations, and other organizations who had been involved in at least one health policy-making process in the Israeli health system in the last five years. |
| 6 | Gollust SE, 2017 | Mutual Distrust: Perspectives From Researchers and Policy Makers on the Research to Policy Gap in 2013 and Recommendations for the Future. | To understand the perspectives of United States policy- makers and health researchers on the barriers and facilitators to translating health evidence into the policy process, with a particular focus on issues related to relationship building. | State-level staffers and legislators who attended annual legislative summit in August 2013 and focused on health and/or human services issues. |
| 7 | Haynes AS, 2011 | Galvanizers, guides, champions, and shields: the many ways that policymakers use public health researchers. | To describe how policymakers use researchers in policymaking and examine how these activities relate to research utilisation models. | Civil servants, ex-premiers, ministers or ex-ministers, ministerial advisers, NGO officers, community group representatives, and independent advocates identified by researcher informants as influential in shaping policy. |
| 8 | Hyder AA, 2011 | National policymakers speak out: are researchers giving them what they need? | To understand the perspectives and attitudes of policymakers towards the use and impact of research in the health sector in low- and middle-income countries | Policymakers working at the national level in governmental ministries, healthcare organizations, and research institutions who have decision-making authority to sign policy documents or allocate funds at the national level. |
| 9 | Jessani NS, 2020 | Exploring the evolution of engagement between academic public health researchers and decision-makers: From initiation to dissolution | To explore the evolution of relationships between academic faculty at one SPH in the US and government decision-makers. | Government decision-makers at city, state, federal, and global levels. |
| 10 | Khan S, 2014 | The Ontario Drug Policy Research Network: Bridging the gap between Research and Drug Policy | To describe the collaboration between the Ontario Drug Policy Research Network (ODPRN) and the Ontario Public Drug Program (OPDP), focusing on ODPRN’s research methodology and unique rapid-response approach for policy engagement. | Policymakers involved in the Ontario Public Drug Programs and the Ontario Drug Policy Research Network. |
| 11 | Langlois EV, 2016 | Enhancing evidence-informed policymaking in complex health systems: Lessons from multi-site collaborative approaches | To reflect on the implementation and impact of two evidence-to-policy approaches implemented in  Low-resource settings. | CoP: Frontline policy/programme managers and implementers; Policy BUDDIES: Subnational policymakers working on chronic non-communicable diseases, nutrition and task shifting for antiretroviral therapy. |
| 12 | Langlois EV, 2019 | Embedding implementation research to enhance health policy and systems: a multi-country analysis from ten settings in Latin America and the Caribbean. | To analyse ten embedded implementation research projects in order to identify barriers and facilitators to embedding research into policy and practice as well as to assess the programme, policy and systems improvements and the cross-cutting lessons in conducting research embedded in real-world policy and systems decision-making. | Decision-makers involved in the embedded implementation research projects. |
| 13 | Loncarevic N, 2021 | Policymakers' research capacities, engagement, and use of research in public health policymaking | To investigate how policymakers’ research capacities influence research use in the health policy process and identify areas where capacity-building interventions give the most meaning and impact. Furthermore, to investigate policymakers’ research engagement and use this to inform public health policy in the public sector in Denmark. | Health policymakers, managers (e.g., hospital and municipality-based managers), and decision-makers from non-governmental organizations who participated in policymaking processes. |
| 14 | Mancuso A, 2021 | Cross-cutting lessons from the Decision-Maker Led Implementation Research initiative. | To describe and summarize the projects, methods, activities, use, and perceived changes resulting from the projects; and to examine the experiences and perceptions of decision-maker-led research by both decision-makers and researchers engaged in the projects to identify enablers and barriers to conducting research and using the results to bring about changes. | Decision-makers who participated as Principal Investigators in the research projects of the DELIR initiative. |
| 15 | Mansilla C, 2017 | The Evidence-Informed Policy Network (EVIPNet) in Chile: lessons learned from a year of coordinated efforts | To describe the experience of implementing the Evidence-Informed Policy Network (EVIPNet) in Chile, including objectives, organizational structure, strategy, activities, main outputs, and evolution over the first year. | Policymakers involved in health policy in Chile. |
| 16 | Mendell J, 2021 | Integrated knowledge translation to strengthen public policy research: a case study from experimental research on income assistance receipt among people who use drugs | To describe the planning, implementation and impact of integrated knowledge translation (IKT) embedded within the study from initiation through to the release of preliminary study findings. | Provincial policymakers from the Ministry of Social Development and Poverty Reduction, the Ministry of Health and the Ministry of Mental Health and Addictions, and senior officials within health authorities. |
| 17 | Mihalicza P, 2018 | Qualitative assessment of opportunities and challenges to improve evidence-informed health policy-making in Hungary - an EVIPNet situation analysis pilot | To present and discuss the findings of the Situation Analysis on policy–research interactions in Hungary and (2) to identify the barriers and opportunities for establishing a KTP within the Hungarian context (role, tasks, organisation). | Individuals working in government agencies, the Ministry of Human Capacities, and various healthcare consultancy and advocacy organizations. |
| 18 | Mijumbi-Deve RM, 2022 | Paper 1: Demand-driven rapid reviews for health policy and systems decision-making: lessons from Lebanon, Ethiopia, and South Africa on researchers and policymakers' experiences. | To appraise the experiences of the three review centers in producing rapid reviews and using findings to enhance evidence-based decision-making for health policy and systems | Policymakers identified by the centers as intimately involved in the production and uptake of rapid reviews. |
| 19 | Mirzoev TN, 2012 | Research-policy partnerships - experiences of the Mental Health and Poverty Project in Ghana, South Africa, Uganda and Zambia. | To analyse experiences of research-policy partnerships between Ministries of Health and research organisations for the implementation of the Mental Health and Poverty Project in Ghana, South Africa, Uganda and Zambia. | Ministry of Health partners in Ghana, South Africa, Uganda, and Zambia. |
| 20 | Onwujekwe O, 2019 | Building the capacity of users and producers of evidence in health policy and systems research for better control of endemic diseases in Nigeria: a situational analysis. | To provide information on the levels of involvement in HPSR+A (among producers of research evidence) and use of research evidence for decision making (among users of evidence) for the control of endemic diseases in two states in Nigeria and highlight potential interventions for improving capacity to undertake and use HPSR+A in policy and decision making. | Policymakers, senior healthcare managers in state MOHs and affiliated health agencies, and data management officers in the MOH in Enugu and Anambra states. |
| 21 | Onwujekwe O, 2020 | Does improving the skills of researchers and decision-makers in health policy and systems research lead to enhanced evidence-based decision-making in Nigeria?-A short term evaluation | To build capacity for HPSR in research scientists, policy/decision-makers and practitioners, and provide knowledge on the level to which they used this capacity in reviewing, re-designing programming, implementing and evaluating strategies and plans to control endemic diseases in two states in southeast Nigeria. | Policymakers from the MOH, programme/project managers, directors in health agencies and parastatals. |
| 22 | Shroff ZC, 2017 | Institutional capacity to generate and use evidence in LMICs: Current state and opportunities for HPSR | To analyse institutional capacity for the generation of HPSR and the use of evidence (including HPSR) more broadly in LMICs. | Policymakers from 24 MOHs in LMICs |
| 23 | Smith N, 2009 | Identifying research priorities for health care priority setting: a collaborative effort between managers and researchers. | To report on a recent effort in British Columbia to have researchers and decision-makers jointly establish an agenda for future research into questions of resource allocation. | Decision-makers from six British Columbia's health authorities |
| 24 | Uneke CJ, 2017 | Improving maternal and child health policymaking processes in Nigeria: An assessment of policymakers' needs, barriers and facilitators of evidence-informed policymaking | To assess the perception of MNCH policymakers regarding their needs and the barriers and facilitators to use of research evidence in policymaking in Nigeria. | Senior staff of various organizations involved in the policy-making process, including the Nigerian Federal Ministry of Health Abuja and its associated ministries, departments and agencies, and the state ministries of health. |
| 25 | Uneke CJ, 2017 | Promoting Researchers and Policymakers Collaboration in Evidence-Informed Policy-Making in Nigeria: Outcome of a Two-Way Secondment Model between University and Health Ministry | To report the outcome of a two-way secondment model between Ebonyi State University (EBSU) and Ebonyi State Ministry of Health (ESMoH) in Nigeria as an innovative collaborative strategy to promote capacity enhancement for evidence-to-policy-to-action. | Senior staff of director cadre in the ESMoH, directly involved with policy-making/implementation process. |
| 26 | Uzochukwu B, 2016 | The challenge of bridging the gap between researchers and policy makers: Experiences of a Health Policy Research Group in engaging policy makers to support evidence informed policy making in Nigeria | To report the experiences of a research group in a Nigerian university when seeking to "do" GRIPP (Gerring research into policy and practice), and the important features and challenges of this process within the African context. | Policymakers and stakeholders involved in selected studies conducted by the HPRG and in a position to influence policy. |
| 27 | Van der Graaf P, 2017 | How do public health professionals view and engage with research? A qualitative interview study and stakeholder workshop engaging public health professionals and researchers | To explore the perspectives of PHPs and researchers when interacting, with a view to identifying barriers to and opportunities for developing practice that is effectively informed by research. | Public Health Professionals |
| 28 | Varallyay NI, 2020 | How does embedded implementation research work? Examining core features through qualitative case studies in Latin America and the Caribbean | To study whether and how key EIR features influence the implementation and outcomes of the research conducted in three distinct settings. | Decision-makers involved in the embedded implementation research projects. |
| 29 | Varallyay NI, 2022 | Strategies to promote evidence use for health programme improvement: learning from the experiences of embedded implementation research teams in Latin America and the Caribbean | To describe strategies study teams used in the post-research phase of EIR to promote evidence-informed programme or policy improvement. | Health Programme and Policy decision-makers from Colombia, the Dominican Republic, and Bolivia who were involved in the iPIER initiative. |
| 30 | Williamson A, 2019 | How are evidence generation partnerships between researchers and policymakers enacted in practice? A qualitative interview study. | To determine why researchers and policymakers choose to work together, how they work together, which partnership models are most common and what the key (1) relationship-based and (2) practical components of successful research partnerships are. | Policy and programme developers and health system decision-makers experienced in working in partnership with researchers. |

CoP Community of Practice; EIR: Embedded Implementation Research; EVIPNet: Evidence-Informed Policy Network; HPSR: Health Policy and Systems Research; iPIER: Improving Program Implementation through Embedded Research; KTP: Knowledge-to-Policy; LMICs: Low- and Middle- Income Countries; MOH: Ministry of Health; PHP: Public Health Professionals; RQ: Research question.
