# Supplementary material for: Enablers and barriers for policymaker engagement in health research from the perspective of policymakers: a scoping review
Source: BMJ Open. 2025 Aug 21;15(8):e099720. doi: 10.1136/bmjopen-2025-099720 (PMC12374644; doi:10.1136/bmjopen-2025-099720)
Supplement: Supplementary file 2 [file bmjopen-2025-099720.reviewer_comments.pdf]

PEER REVIEW HISTORY

BMJ Open publishes all reviews undertaken for accepted manuscripts. Reviewers are asked to complete a checklist review form and are provided with free text boxes to elaborate on their assessment. These free text comments are reproduced below.

This paper was submitted to another journal from BMJ but declined for publication following peer review. The authors addressed the reviewers' comments and submitted the revised paper to BMJ Open. The paper was subsequently accepted for publication at BMJ Open.

ARTICLE DETAILS

Title (Provisional)

Enablers and barriers for policymaker engagement in health research from the perspective of policymakers: a scoping review

Authors

Guerrero-Torres, Lorena; Ismail, Anas; Savedoff, William; Sheikh, Kabir; Schleiff, Meike

---

VERSION 1 - REVIEW

**Reviewer**	**1**
**Name**	**Reviewer 1**
**Affiliation**	
**Date**	**30-Dec-2024**
**COI**	**No competing interest**

Comments not published pending reviewer's consent

**Reviewer**	**2**
**Name**	**Reviewer 2**
**Affiliation**	
**Date**	**30-Dec-2024**
**COI**	**No competing interest**

Comments not published pending reviewer's consent

VERSION 2 - REVIEW

**Reviewer**	**3**
**Name**	**Wettermark, Björn**
**Affiliation**	**Uppsala Universitet**
**Date**	**20-Apr-2025**
**COI**	**None**

General comments

Thank you for the opportunity to read this interesting study. I was not reviewer for the original submission and hereby comment on the version that has already been reviewed once. I read through the comments from the previous reviewers and feel that the authors in general have addressed them well, but I have some further questions as presented below.

Specific comments for revision

Title & general: The addition of “national and subnational” to the title in the last review does not add to clarity, but the word “health” is of course important. There is certain challenge with the word “subnational” given that health systems differ substantially between countries. In some cases “subnational” may refer to a region which act as a country, in other cases it may refer to individual healthcare professionals. I would suggest to omit this and instead expand a bit on the term “policymaker”. It currently comes in the method section, but it could be worthwhile to elaborate about it already in the introduction and how this is considered in this specific paper.

Abstract: While the aim is to present both enablers and barriers, no barriers are mentioned in the abstract. The last sentence in the abstract “We offer practical ways to enhance research-policy…” could be omitted since it goes beyond what was done on the paper.

Introduction: the addition of the sentence in the review ”to help develop more effective strategies that facilitate meaningful collaboration and ensure that research is responsive to policy needs.” is a bit to go beyond what was actually done in the review, given which studies that were reviewed. I suggest to omit it or move to discussion.

Introduction, abstract and method: The fact that studies were excluded if they did not include policymakers, where engagement was passive, or perspectives were marginal or not clearly outlined, is problematic since it might increase the risk of missing important studies. Could this be reflected on?

Methods: This section is in general well written and complements for using the standardized tools. Explaining it with “PICO” does not that well match the eclectic type of studies included in the review though. Especially what is considered as “O”. There are other acronyms that are more suitable for these type of questions. One option might be “SPICE”. Formulating

Questions for Evidence-Based Practice (SPICE Framework) | Resource Details | National Collaborating Centre for Methods and Tools

One concrete thing to clarify in the inclusion/exclusion criteria is that qualitative studies are included. These are not mentioned in the supplement of study designs.

Method: The time period for the search starts 2007 referring to "Sound Choices" flagship report Would be good to elaborate more on this and why this is a suitable milestone, then preferably move to introduction.

Results and discussion: I agree with one of the previous reviewers that it is surprising with the few studies. Also the uneven distribution by countries. A clear example also the disproportionally large number of papers from Nigeria. I feel that the risk for publication bias is high, which could be commented further.

Results: It would have been valuable to add a column with the study design in the first table, given that different designs provide very different evidence.

Discussion and/or results: I lack some more critical appraisal of the included papers. What is the quality of the evidence

Discussion and key messages: It is claimed that this review bring in experiences from the COVID-19 pandemic. It is of course interesting given the explosion of policy decisions during covid. There rare also challenges with it with the "covidization of research" that did not add much value. If bringing in this, it needs a more developed discussion on Covid, referring to other papers in the scientific debate on Covid and policy decisions.

Discussion: Early in the introduction, this paper outlines the novely being the focus on the policymakers views on contrast to the researchers point of view. It would be nice to bring this back a bit more in the discussion, also adding layers on different types of policymakers.

**Reviewer**	**4**
**Name**	**Reyes-Santías, Francisco**
**Affiliation**	**Universidad de Vigo, Organización de Empresas e Mercadotecnia**
**Date**	**17-May-2025**
**COI**	**None**

REVIEW REPORT FOR THE STUDY "ENABLERS AND BARRIERS FOR NATIONAL AND SUBNATIONAL POLICYMAKER ENGAGEMENT IN HEALTH RESEARCH FROM THE PERSPECTIVE OF POLICYMAKERS: A SCOPING REVIEW"

Journal: BMJ

The paper "Enablers and barriers for national and subnational policymaker engagement in health research from the perspective of policymakers: a scoping review" performs a scoping review that identifies organizational factors like institutional partnerships and clear roles as key enablers of policymaker engagement in health research, based on analysis of 30 studies. While offering practical insights for evidence-based policymaking, the findings primarily reflect national-level perspectives and could benefit from broader geographic representation.

Title and summary. Title: Clear but slightly verbose; could be more concise while maintaining key elements (policymaker perspective, national/subnational focus, scoping review method). The abstract is well-structured with comprehensive methods and findings, but would benefit from a brief mention of the geographic distribution of studies and 1-2 key barriers for balance (e.g., institutionalized partnerships, clear roles, expert advice from researchers). Overall, effective communication of study purpose and outcomes.

Structure of the article. The contents are well organized, and they adhere to the IMRaD structure. It includes a theoretical framework of the research problem.

Strengths of the Study

1. Clear and Focused Research Objective: The study effectively addresses a critical gap in the literature by focusing on policymakers' perspectives (rather than researchers') regarding research engagement. This is novel and policy-relevant. Also, the distinction between national and subnational policymakers adds depth to the analysis.

2. Methodology: The scoping review approach is appropriate for mapping the breadth of evidence on this topic. Also, it shows a comprehensive search strategy, including multiple databases (MEDLINE, Cochrane, grey literature) and clear inclusion/exclusion criteria. Moreover, the use of MAXQDA for qualitative coding and thematic analysis strengthens data reliability, and the Multi-level analysis (individual, organizational, contextual) provides a structured way to categorize enablers/barriers.

3. Relevant and Practical Findings: The study highlights organizational factors (e.g., institutional partnerships, formal agreements) as the most critical enablers, useful for real-world implementation, and identifies actionable strategies (e.g., defining roles, leveraging networks) that could improve research-policy engagement.

4. Transparent Reporting: Clear documentation of the screening aligns with PRISMA-ScR guidelines and acknowledges temporal trends, suggesting growing policy interest in this area.

Critiques and Limitations

1. Geographic and Income-Level Bias: The review does not specify whether included studies represent diverse global settings (e.g., low vs. high-income countries). Moreover, policymaker engagement barriers may differ significantly in resource-constrained systems (e.g., lack of institutional capacity vs. political interference).

2. Underrepresentation of Subnational Policymakers: The conclusion notes that most studies focused on national-level engagement and subnational policymakers (e.g., local health departments) may face different challenges (e.g., limited funding, less access to research).

4. Limited Discussion of Power Dynamics: The review does not deeply explore political influences (e.g., lobbying, electoral cycles) that may override evidence-based decision-making. Also, the term "Supportive institutions" is vague (what specific policies or leadership actions enable engagement?)

5. Weaknesses in Practical Recommendations: The conclusion offers broad suggestions (e.g., "enhance collaboration") but lacks a concrete toolkit or checklist for policymakers/researchers, and authors could propose specific interventions (e.g., mandatory research-policy liaison roles in health ministries).

Overall, the study is well-conducted and the study is methodologically sound, and addresses an important gap, but would benefit from a stronger theoretical foundation, some more nuanced analysis of political and subnational contexts, and clearer policy recommendations. Publish with Minor Revisions.

VERSION 2 - AUTHOR RESPONSE

<u>Reviewer 3</u>

General comments - Thank you for the opportunity to read this interesting study. I was not reviewer for the original submission and hereby comment on the version that has already been reviewed once. I read through the comments from the previous reviewers and feel that the authors in general have addressed them well, but I have some further questions as presented below.

1. Specific comments for revision - Title & general: The addition of "national and subnational" to the title in the last review does not add to clarity, but the word "health" is of course important. There is certain challenge with the word "subnational" given that health systems differ substantially between countries. In some cases "subnational" may refer to a region which act as a country, in other cases it may refer to individual healthcare professionals. I would suggest to omit this and instead expand a bit on the term "policymaker". It currently comes in the method section, but it could be worthwhile to elaborate about it already in the introduction and how this is considered in this specific paper.

Response: Thank you for this valuable comment. We agree and have removed the term "national and subnational" from the title, which is now *"Enablers and barriers for policymaker engagement in health research from the perspective of policymakers: a scoping review".* On your comment related to policymakers, we completely agree with the need to expand on this matter. We have added the following sentences in the methods section to keep the introduction concise and focused on the rationale for this work, and reserve operational definitions and rationale for these in the appropriate section, as consistent with the JBI Manual (Chapter related to Scoping Reviews).

Policymakers are a heterogeneous group whose roles, responsibilities and influence vary depending on governance structures, health system organization and political context. For the purposes of this review, we included studies that involved policymakers, decision-makers, or regulators at any level (national or sub-national). We adopted the definition of Deverka et al. for policymakers and regulators: "individuals and organizations that create, monitor and oversee policies or regulations of healthcare-related issues, such as federal, state and local government agencies, medical and professional organizations and clinical guidelines developers" [14]. This definition acknowledges that policy and decision-making not only occur within government, but also in other professional bodies and recognizes the diversity of these actors across national and subnational contexts. From here on, we use "policymakers" to refer to policymakers, decision-makers and regulators.

2. Abstract: While the aim is to present both enablers and barriers, no barriers are mentioned in the abstract. The last sentence in the abstract "We offer practical ways to enhance research-policy…" could be omitted since it goes beyond what was done on the paper.

Response: Thank you for noting this in the abstract. We have included the three most frequent barriers in the results section and removed the final sentence as suggested.

The most frequent barriers were the lack of regulations, infrastructure, funding and communication channels to support engagement; the lack of skills of researchers to understand policymaking processes and work in collaboration with policymakers; and the mismatch in priorities, values, perspectives and expectations.

3. Introduction: the addition of the sentence in the review "to help develop more effective strategies that facilitate meaningful collaboration and ensure that research is responsive to policy needs." is a bit to go beyond what was actually done in the review, given which studies that were reviewed. I suggest to omit it or move to discussion.

Response: Thank you for highlighting this point. We agree that the original sentence could be interpreted as suggesting that our review itself developed new strategies, which was not the case. We have revised the sentence to clarify that our review aimed to inform the development of such strategies, rather than implying that we developed them ourselves.

In conducting this study, we attempt to fill this gap in the literature to inform the development of more effective strategies that facilitate meaningful collaboration and ensure that research is responsive to policy needs.

4. Introduction, abstract and method: The fact that studies were excluded if they did not include policymakers, where engagement was passive, or perspectives were marginal or not clearly outlined, is problematic since it might increase the risk of missing important studies. Could this be reflected on?

Response: We appreciate the opportunity to clarify this point. Our review aimed to focus specifically on studies that included the perspectives of policymakers themselves, rather than studies reporting only on researcher or third-party perspectives. While we acknowledge that this inclusion criterion might have led to the exclusion of some studies where engagement was more limited or passive, studies characterized by passive or minimal engagement, such as those involving only the presentation of research findings to policymakers without active involvement, would not have offered the necessary insights to understand policymakers' own experiences, perceptions, and reflections on their engagement in research processes. Including such studies could have confounded our analysis by mixing researcher-driven perspectives with those of policymakers themselves. By focusing on studies that directly captured policymakers' views, we sought to ensure that our synthesis authentically reflected their experiences and perspectives, which was essential to reaching the objective of our study.

We have included the sentence below in the discussion section to acknowledge this potential limitation.

Furthermore, by focusing on studies that include the perspectives of policymakers who engaged in the research process, we acknowledge that studies we excluded, due to with limited or passive policymaker engagement, might have offered additional insights that our sample did not address.

5. Methods: This section is in general well written and complements for using the standardized tools. Explaining it with "PICO" does not that well match the eclectic type of studies included in the review though. Especially what is considered as "O". There are other acronyms that are more suitable for these type of questions. One option might be "SPICE". Formulating Questions for Evidence-Based Practice (SPICE Framework) | Resource Details | National Collaborating Centre for Methods and Tools

Response: Thank you for your valuable suggestions. We acknowledge that the PICO framework is typically applied in systematic reviews of clinical interventions and may be less ad hoc for the types of studies included in this review. However, the PICO framework offered a structured and systematic way to organize our criteria, with us (the research team) adapting this framework to the needs of our study. We recognize that other frameworks could have been appropriate, and we will take this recommendation into account for future reviews on similar topics.

6. One concrete thing to clarify in the inclusion/exclusion criteria is that qualitative studies are included. These are not mentioned in the supplement of study designs.

Response: Thank you for highlighting this point. Qualitative studies were included in our scoping review, as reflected in the descriptive nature of the studies analyzed and presented. Although we cannot modify the supplement since it is part of our pre-established protocol, we have clarified in the methods section of the manuscript that qualitative studies were included.

Our review focused on studies that captured the enablers and barriers to policymaker engagement in research from the perspective of policymakers, including qualitative studies.

7. Method: The time period for the search starts 2007 referring to "Sound Choices" flagship report Would be good to elaborate more on this and why this is a suitable milestone, then preferably move to introduction.

Response: Thank you for your comment. The "Sound Choices: Enhancing Capacity for Evidence-Informed Health Policy" flagship report was a key publication advocating for the integration of research (health policy and systems research, in particular) into decision-making processes. This report highlighted the important role of policymakers in translating research evidence into actionable policies and led to a vast amount of research on evidence-to-policy strategies, including how to engage with policymakers. For this reason, we chose 2007 as the starting point of our review to capture studies that built upon these foundational principles and frameworks. We have added the following sentence into our manuscript (methods section):

We restricted our search to articles in English, published from 2007 onwards, coinciding with the publication of the "Sound Choices" flagship report of the Alliance for Health Policy and Systems Research [3], which catalyzed efforts to integrate research into decision-making processes and emphasized the importance of engaging policymakers.

8. Results and discussion: I agree with one of the previous reviewers that it is surprising with the few studies. Also the uneven distribution by countries. A clear example also the disproportionally large number of papers from Nigeria. I feel that the risk for publication bias is high, which could be commented further.

Response: Thank you for this important comment. It may be worth to mention that Nigeria is a recognized country for strong institutions and researchers in the field of health policy and systems research and evidence-to-policy approaches, which may partially explain the high volume of publications from this country. However, we acknowledge that the distribution of studies across countries may reflect not only the underlying research activity but also the availability of studies in English and differences in publication practices. We have added the following sentences in response to your comments.

We also acknowledge that our selection criteria resulted in an uneven distribution of articles; especially regarding geographical representativity. To some degree, this is a natural consequence of finding few studies, which may reflect the limited number of policymaker engagement experiences, along with a low probability of having experiences documented in the public record. The articles may also exhibit publication bias, with authors and journals willing to report successful cases and reticent about reporting failed experiences. Limiting the sample to publications in English may also have excluded worthwhile experiences that might have been documented in other languages.

9. Results: It would have been valuable to add a column with the study design in the first table, given that different designs provide very different evidence.

Response: Thank you for this valuable comment. While we agree that including the study design would have provided this information to readers, we found that most of the included studies used

descriptive designs relying on qualitative or mixed-methods, such as interviews, surveys and document reviews. Therefore, we decided not to add a separate column to the table. However, in response to your important comment, we have now added a sentence related to this in the results section to clarify.

As described in Table 1, the study settings of most articles were national (n=18, 60%) and subnational (n=8, 27%). Most studies (n=29, 97%) included in this review employed descriptive designs relying on qualitative or mixed-methods, such as interviews, surveys, and document reviews. The number of policymakers included as participants in the studies varied widely from 7 to 600. Half of the studies engaged policymakers in several phases of the research process and used different engagement methods.

10. Discussion and/or results: I lack some more critical appraisal of the included papers. What is the quality of the evidence

Response: Thank you for your comment. Our scoping review follows the recommendations on the JBI Manual for Evidence Synthesis, which explains that, unlike a systematic review, scoping reviews do generally perform methodological appraisal to determine the quality of the evidence, as scoping reviews aim to provide an overview or map the evidence. Given this, we have added the following limitations in the "Strengths and limitations" section after the abstract. We hope this addresses your concern.

We did not appraise the methodological quality of included studies, however, this is consistent with the scoping review design and results of this study should be interpreted as an overview of the evidence.

11. Discussion and key messages: It is claimed that this review bring in experiences from the COVID-19 pandemic. It is of course interesting given the explosion of policy decisions during covid. There rare also challenges with it with the "covidization of research" that did not add much value. If bringing in this, it needs a more developed discussion on Covid, referring to other papers in the scientific debate on Covid and policy decisions.

Response: Thank you for your comment. While acknowledging the relevance of the COVID-19 pandemic to the policymaker engagement process, this review focuses on broader, generalizable enablers and barriers to policymaker engagement in research, without concentrating specifically on the pandemic context. Therefore, a detailed discussion on COVID-19-related research or policymaking would be outside the scope of this review.

12. Discussion: Early in the introduction, this paper outlines the novely being the focus on the policymakers views on contrast to the researchers point of view. It would be nice to bring this back a bit more in the discussion, also adding layers on different types of policymakers.

Response: Thank you for this comment. We have added the following lines to the discussion section in response to your suggestion on the different types of policymakers:

This scoping review captures the views of a diverse range of policymakers, from national and subnational health ministry officials and health program managers to senior policymakers from ministries of finance, social development, and other key sectors (Supplementary File, Appendix F). While we did not specifically analyze the different perspectives by type or level of policymaker, their roles and responsibilities within their own institutions may also influence their engagement in the research process.

<u>**Reviewer 4**</u>

1. The paper "Enablers and barriers for national and subnational policymaker engagement in health research from the perspective of policymakers: a scoping review" performs a scoping review that identifies organizational factors like institutional partnerships and clear roles as key enablers of policymaker engagement in health research, based on analysis of 30 studies. While offering practical insights for evidence-based policymaking, the findings primarily reflect national-level perspectives and could benefit from broader geographic representation.

Response: Thank you for this comment. We agree that many of the studies (60%) in this scoping review reflect national-level perspectives, which may limit generalizability of our findings to subnational or contexts not represented here. Given the specific focus of our study (policymakers' perspective in the engagement of research), and our inclusion/exclusion criteria (i.e. English language studies, active engagement of policymakers, etc.), the included studies were primarily from specific countries or regions where such research has been conducted and published. We recognize that including studies from a broader range of countries would improve the generalizability of our findings. However, we did not find such studies thorough our comprehensive search strategy.

2. Title and summary. Title: Clear but slightly verbose; could be more concise while maintaining key elements (policymaker perspective, national/subnational focus, scoping review method). The abstract is well-structured with comprehensive methods and findings, but would benefit from a brief mention of the geographic distribution of studies and 1-2 key barriers for balance (e.g., institutionalized partnerships, clear roles, expert advice from researchers). Overall, effective communication of study purpose and outcomes. Structure of the article. The contents are well organized, and they adhere to the IMRaD structure. It includes a theoretical framework of the research problem.

Response: Thank you for your positive comments. We have revised the title and abstract slightly in response to previous comments (i.e. removed subnational/national from title, added barriers for balance). We hope this addresses your concerns.

3. Strengths of the Study
1. Clear and Focused Research Objective: The study effectively addresses a critical gap in the

literature by focusing on policymakers' perspectives (rather than researchers') regarding research engagement. This is novel and policy-relevant. Also, the distinction between national and subnational policymakers adds depth to the analysis.
2. Methodology: The scoping review approach is appropriate for mapping the breadth of evidence on this topic. Also, it shows a comprehensive search strategy, including multiple databases (MEDLINE, Cochrane, grey literature) and clear inclusion/exclusion criteria. Moreover, the use of MAXQDA for qualitative coding and thematic analysis strengthens data reliability, and the Multi-level analysis (individual, organizational, contextual) provides a structured way to categorize enablers/barriers.
3. Relevant and Practical Findings: The study highlights organizational factors (e.g., institutional partnerships, formal agreements) as the most critical enablers, useful for real-world implementation, and identifies actionable strategies (e.g., defining roles, leveraging networks) that could improve research-policy engagement.
4. Transparent Reporting: Clear documentation of the screening aligns with PRISMA-ScR guidelines and acknowledges temporal trends, suggesting growing policy interest in this area.

Response: Thank you. We appreciate your positive comments and feedback on our study.

4. Critiques and Limitations

1. Geographic and Income-Level Bias: The review does not specify whether included studies represent diverse global settings (e.g., low vs. high-income countries). Moreover, policymaker engagement barriers may differ significantly in resource-constrained systems (e.g., lack of institutional capacity vs. political interference).

Response: Thank you for this comment. We acknowledge this gap in our analysis. Upon revision of our data, we found that 13 of our studies focused on LMICs (e.g. Nigeria, Iran, Mozambique), 13 on HICs (e.g. Israel, Canada, Australia), and 4 studies included both LMIC and HIC settings (e.g. studies covering Argentina, Chile, Peru, Bolivia, and Mexico) and global in scope without country-level differentiation. While this diversity reflects the breadth of policymaker engagement experiences across different governance structures, health system organizations, and political contexts, our analysis did not focus on comparing perspectives across these contexts. We acknowledge this limitation and suggest that future research could explore these differences in more detail. In response to this comment, we have added the following sentences in the limitations section:

Our review included high-, middle-, and low-income countries, as well as experiences that were global, regional, and local. However, we did not disaggregate our findings along these dimensions due to the small number of studies. Consequently, this study is limited in what it can say about differences in enablers and barriers across such contexts.

5. Critiques and Limitations – Underrepresentation of Subnational Policymakers: The conclusion notes that most studies focused on national-level engagement and subnational policymakers (e.g., local health departments) may face different challenges (e.g., limited funding, less access to research).

Response: Thank you for this comment. We agree that findings from national-level settings may not reflect the realities of subnational contexts. While our review did include subnational studies (n=8, 27%), we did not disaggregate data to allow for a detailed analysis of the differences between national and subnational contexts. As noted above, we included a sentence related to this in the limitation section.

6. Critiques and Limitations – Limited Discussion of Power Dynamics: The review does not deeply explore political influences (e.g., lobbying, electoral cycles) that may override evidence-based decision-making. Also, the term "Supportive institutions" is vague (what specific policies or leadership actions enable engagement?)

Response: Thank you for noting this important issue. We recognize that political factors such as lobbying, and electoral cycles can influence how and when policymakers engage with research. However, the scope of this review was limited to examining enablers and barriers to engagement and we did not explore the broader political economy factors shaping policymaker engagement. Regarding the term "supportive institutions," we used it in line with the descriptions provided in the reviewed studies, where it generally referred to organizational arrangements or mechanisms that enable research-policy engagement, such as dedicated research units or formal agreements. We agree that future research could address these issues, including the role of political dynamics and specific institutional actions that support evidence-based policymaking.

7. Critiques and Limitations – Weaknesses in Practical Recommendations: The conclusion offers broad suggestions (e.g., "enhance collaboration") but lacks a concrete toolkit or checklist for policymakers/researchers, and authors could propose specific interventions (e.g., mandatory research-policy liaison roles in health ministries).

Response: Thank you for this important comment. Our review aimed to inform the development of strategies that could improve the engagement between researchers and policymakers in research processes. Developing concrete recommendations or toolkits would require a different type of study beyond the scope of this review. However, we did include a comment on this in our manuscript: "In addition, various tools, checklists, and recommendations to improve this engagement already exist [4,6,27,28] ." Future research could build on our study to develop more practical tools for policymakers and researchers, including those based on specific contexts.

8. Critiques and Limitations - Overall, the study is well-conducted, and the study is methodologically sound, and addresses an important gap, but would benefit from a stronger theoretical foundation, some more nuanced analysis of political and subnational contexts, and clearer policy recommendations. Publish with Minor Revisions.

Response: Thank you for this comment and for recognizing the methodological rigor of our study. We appreciate your suggestion on the theoretical foundation and the need for analyzing political and subnational contexts to provide specific recommendations. However, as noted previously, providing specific policy recommendations is outside of the scope and nature of scoping reviews. The aim of scoping reviews is rather to provide an overview of the evidence or to answer questions

related to the diversity of the evidence available. We hope this clarifies your concerns and agree that future studies could consider this as a focus.

VERSION 3 - REVIEW

**Reviewer**	**3**
**Name**	**Wettermark, Björn**
**Affiliation**	**Uppsala Universitet**
**Date**	**12-Jul-2025**
**COI**	

I still consider PICO not to be the best structure for this type of research question, but I acknowledge the authors unwillingness to change since it was a pre-specified protocol.

Apart from that, I find that the authors have well addressed all the comments raised in my review.
